# Treed Gaussian processes for animal movement modeling

**DOI:** 10.1002/ece3.11447

**Published:** 2024-06-02

**Authors:** Camille J. Rieber, Trevor J. Hefley, David A. Haukos

**Affiliations:** ^1^ Department of Statistics and Kansas Cooperative Fish and Wildlife Research Unit Kansas State University Manhattan Kansas USA; ^2^ Department of Statistics Kansas State University Manhattan Kansas USA; ^3^ U.S. Geological Survey, Kansas Cooperative Fish and Wildlife Research Unit Kansas State University Manhattan Kansas USA

**Keywords:** Bayesian model, lesser prairie‐chicken, machine learning, movement modeling, population‐level inference, telemetry, treed Gaussian processes, wildlife management

## Abstract

Wildlife telemetry data may be used to answer a diverse range of questions relevant to wildlife ecology and management. One challenge to modeling telemetry data is that animal movement often varies greatly in pattern over time, and current continuous‐time modeling approaches to handle such nonstationarity require bespoke and often complex models that may pose barriers to practitioner implementation. We demonstrate a novel application of treed Gaussian process (TGP) modeling, a Bayesian machine learning approach that automatically captures the nonstationarity and abrupt transitions present in animal movement. The machine learning formulation of TGPs enables modeling to be nearly automated, while their Bayesian formulation allows for the derivation of movement descriptors with associated uncertainty measures. We demonstrate the use of an existing R package to implement TGPs using the familiar Markov chain Monte Carlo algorithm. We then use estimated movement trajectories to derive movement descriptors that can be compared across individuals and populations. We applied the TGP model to a case study of lesser prairie‐chickens (*Tympanuchus pallidicinctus*) to demonstrate the benefits of TGP modeling and compared distance traveled and residence times across lesser prairie‐chicken individuals and populations. For broad usability, we outline all steps necessary for practitioners to specify relevant movement descriptors (e.g., turn angles, speed, contact points) and apply TGP modeling and trajectory comparison to their own telemetry datasets. Combining the predictive power of machine learning and the statistical inference of Bayesian methods to model movement trajectories allows for the estimation of statistically comparable movement descriptors from telemetry studies. Our use of an accessible R package allows practitioners to model trajectories and estimate movement descriptors, facilitating the use of telemetry data to answer applied management questions.

## Graphical abstract

We apply a recently developed Bayesian machine learning model to both increase the accessibility of complex animal movement models and simultaneously achieve advanced modeling of highly varied telemetry data. By nesting this model within a widely applicable inferential framework and utilizing an accessible R package for modeling, we facilitate application by practitioners to model trajectories, estimate movement descriptors, and answer applied management questions.

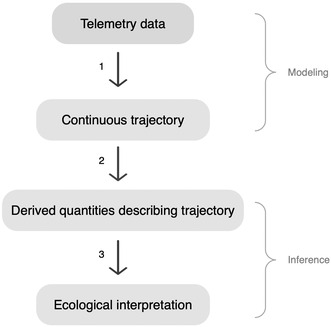

## 1 INTRODUCTION

Telemetry studies are ubiquitous in wildlife ecology for investigating the interactions of individuals with their environments. Wildlife telemetry data consist of multiple locations of a tagged animal recorded over time; these data are now extremely prevalent and accessible across taxa. Technological advancements, such as the use and improvement of Global Positioning System (GPS) satellite transmitters, enable researchers to record locations at finer temporal resolution with greater spatial certainty. While telemetry data recorded by such tools hold information for researchers to explore a wide range of topics (e.g., space use, resource selection, barrier identification), modeling animal movements and achieving useful inferences from telemetry data remain challenging. Available models often pose barriers to use due to the advanced statistical modeling required for their implementation (Patterson et al., 2017). Because telemetry studies hold the potential to answer a multitude of management and research questions, it is important to improve methods for extracting information from telemetry data. Advancements in Bayesian modeling and machine learning techniques provide promising possibilities for modeling animal movement while lowering practical barriers to application and inference.

**FIGURE 1:**
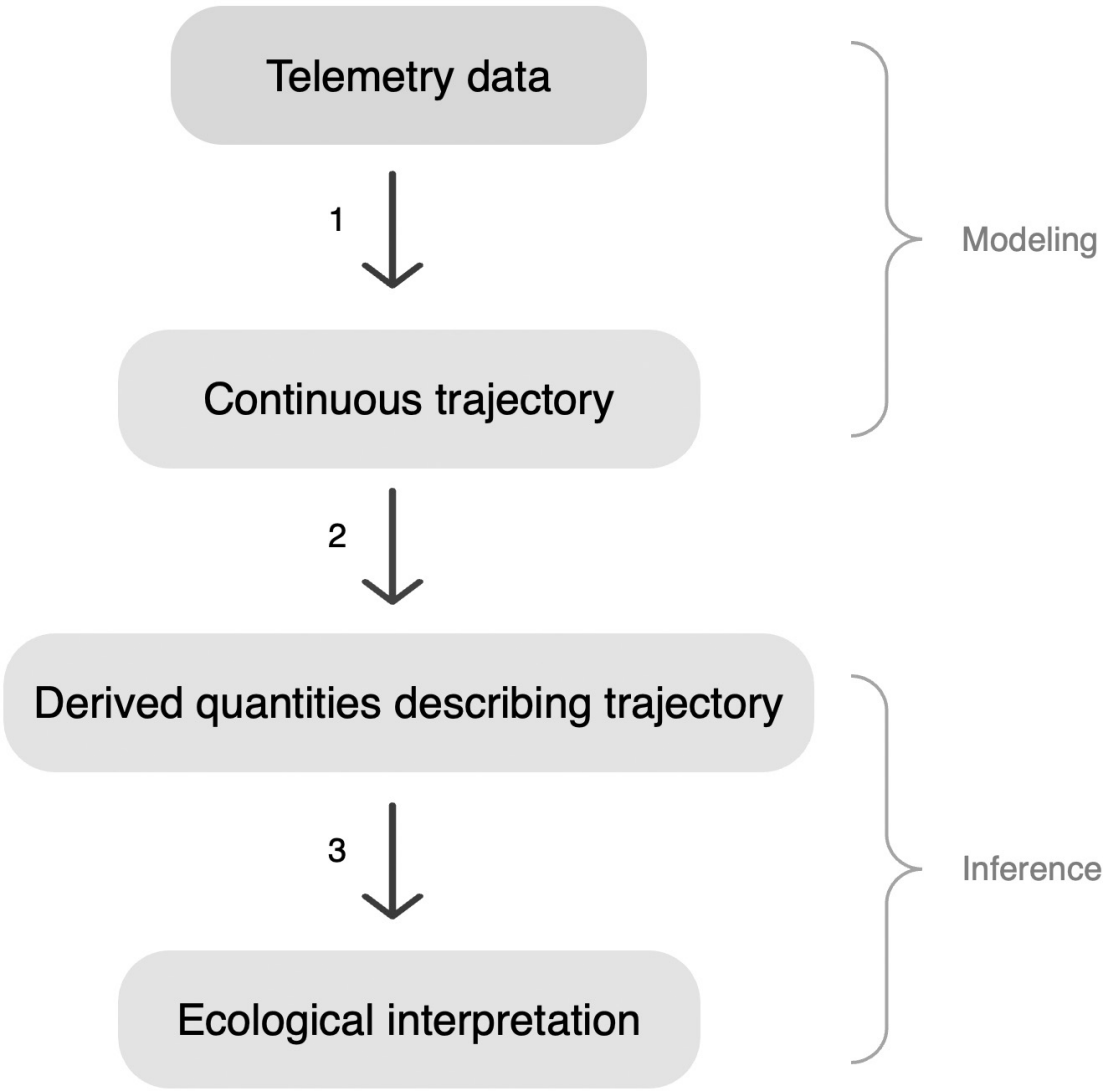
Summary of the animal movement analysis framework. Step 1 fits a model to telemetry data and uses it to predict locations across the study period, thus estimating a continuous movement trajectory. Step 2 makes inference about movement by estimating statistics describing the trajectory (e.g., average meters per hour). In step 3, researchers and managers interpret these values to evaluate hypotheses and guide management.

Telemetry data are typically comprised of locations recorded at discrete time points at a predetermined time interval (e.g., every 30 min, hourly, etc.) with possible irregularity of interval length due to missing data. To predict an animal's location between data points, researchers can estimate the likely trajectory of the animal's movement (Hooten et al., 2017). Using the estimated trajectories, researchers then derive descriptive statistics to summarize movement characteristics such as distance traveled, speed, and directionality (Johnson et al., 2011). This summarization of trajectories can be used to answer countless ecological questions, such as comparing how translocated individuals select habitats (e.g., Picardi et al., 2022) or the distance at which anthropogenic infrastructure affects animal behaviors (e.g., Londe et al., 2022). Using statistical models to estimate trajectories and then summarizing trajectories to answer research questions is a widely used inferential framework and can be visualized in Figure 1.

The first step of the inferential framework is to estimate the movement trajectory of individual animals. There are multiple movement models available to accomplish this, each with their own benefits (Patterson et al., 2017). The simplest approach is to draw straight lines connecting recorded telemetry points (e.g., Londe et al., 2022). Although this method continues to be implemented and useful in wildlife ecology, it is now common practice to fit a continuous statistical model, often in the form of a Gaussian process (GP), to recorded telemetry points (Hooten et al., 2017). This allows for model‐based prediction of an animal's location at any time, thus estimating a continuous‐time movement trajectory (Johnson et al., 2008). Many of these models utilize a Bayesian formulation to provide quantified uncertainty on the estimated trajectory (Johnson et al., 2011).

Most continuous‐time animal movement models perform inference by providing estimations and interpretations of parameters describing the modeled movement trajectory (e.g., Hanks et al., 2011). However, these parameters may not always target the desired inference, especially when the model is used to address multiple management or ecological questions. If the goal of modeling a trajectory is to estimate statistics that can be interpreted for specific applied purposes, our suggested inferential framework (Figure 1) emphasizes that prediction of a trajectory, not model parameters, should be the focus of modeling. In addition to the possible misalignment of parameters and inferential goals, parametric models can be time‐consuming to construct and are often specific to individual studies (Wijeyakulasuriya et al., 2020).

Alternatively, we present a prediction‐focused approach to modeling animal movement, as was recommended by Wijeyakulasuriya et al. (2020). Accurate prediction of unknown locations is required to reconstruct, and thus draw inference from, animal movement trajectories (Figure 1, step 1). Machine learning offers this accuracy, coupled with speed and ease of implementation (Wijeyakulasuriya et al., 2020). While applying a machine learning model, we utilize Bayesian methods to quantify the uncertainty of trajectory estimates. Both machine learning and Bayesian methods are common in wildlife ecology, though Bayesian machine learning remains underutilized, especially in practical applications (Tuia et al., 2022). We implemented a novel application of treed Gaussian process (TGP) models, a recently developed Bayesian machine learning technique, to animal movement modeling (Gramacy, 2005). Applying this phenomenological machine learning model to movement data essentially automates trajectory estimation and frees practitioners from model construction and customization.

As well as increasing ease of implementation and facilitating inference, our proposed model addresses an additional issue intrinsic to telemetry data. Regardless of their formulation, continuous‐time animal movement models must account for the high variability of animal movement. It is intuitive that animals change their movement patterns at distinct times and often abruptly (Wolfson et al., 2022). Data produced by such a nonstationary process lack a consistent statistical pattern across time and present challenges to most modeling approaches. While hidden Markov models (Morales et al., 2004) explicitly model these transitions, they are most commonly implemented in discrete time. There are benefits to modeling movement in continuous time (McClintock et al., 2014), and current research explores methods to model the temporal‐heterogeneity of animal movement in continuous time. Recent methods include temporal warping, continuous‐time hidden Markov models, or time‐varying differential equations (Glennie et al., 2023; Hooten & Johnson, 2017; Michelot et al., 2021). However, these continuous‐time approaches can be complex and require study‐specific formulation, and thus are often preventative to implementation by practitioners.

We propose that the nonstationary nature of animal movement be modeled by TGPs, an extension of the commonly used GP model. Treed partitioning has an intuitive, yet previously underutilized, application to animal movement. Animals move in different ways for distinct periods of time, with discontinuities that naturally suggest a divide‐and‐conquer modeling apparatus. TGP modeling allows different movement patterns to have different parameterizations, fitting the model more precisely while also accounting for nonstationarity. TGP models have strong predictive power and increase the already highly accurate prediction of GP models, aligning with our goal of predicting trajectories from telemetry data (Gramacy, 2020; Gramacy & Lee, 2008). The combination of the traditional GP model and the machine learning partitioning implemented by TGPs offers an accurate, efficient, and easily implemented model for nonstationary and large telemetry data, while the Bayesian formulation of TGPs allows uncertainty measures to be propagated throughout computations. In addition to the benefits provided by the modeling capabilities of TGPs, Gramacy (2007) developed a user‐friendly and efficient R package for TGP modeling. The tgp package implements Bayesian modeling of TGPs via Markov chain Monte Carlo (MCMC) integration, which is beneficial to practitioners familiar with MCMC algorithms.

We leveraged the accessibility of the tgp package in R to apply Bayesian machine learning models to animal movement while simultaneously providing a novel and intuitive method for modeling the nonstationarity of animal telemetry data. We outline the statistical background for Bayesian continuous‐time animal movement models, TGPs, and estimating comparable statistics from these modeled trajectories. Additionally, we demonstrate how our framework performs population‐level inference by estimating statistics describing groups of trajectories. While covering these topics, we maintain the accessibility of TGP modeling to non‐statisticians. Appendix S1 provides a guide to the statistical underpinnings for TGP movement models for interested readers, but it is not required for use of the tgp R package. To demonstrate the novel application of TGPs to wildlife telemetry data, we use a case study of lesser prairie‐chicken (*Tympanuchus pallidicinctus*) GPS telemetry data. To facilitate use by practitioners on their own datasets, a guide to the implementation of TGP movement modeling in R is provided in Appendix S2. This paper and its supporting information aim to both improve the applicability and accessibility of Bayesian continuous‐time animal movement modeling, while simultaneously addressing practical modeling challenges posed by telemetry data.

## 2 MOVEMENT MODELING

### 2.1 Inferential framework

The general paradigm of statistical inference within wildlife ecology is to specify a model, fit it to the collected data, and obtain estimates (with uncertainty quantification) of model parameters. These parameters are then interpreted for the system of interest. Though this paradigm may be applied to inference from telemetry data, we instead follow the framework outlined in Figure 1. Telemetry data are collected and used to estimate the parameters of the movement model, but these parameters are not used for inference. Instead, the fitted model is used for prediction of the complete trajectory traversed by the animal, and relevant inferences are then derived from the estimated movement trajectory. This general estimation of trajectories between recorded telemetry points is common throughout the literature (e.g., Buderman et al., 2016; Johnson et al., 2011).

### 2.2 Continuous‐time movement models

**FIGURE 2:**
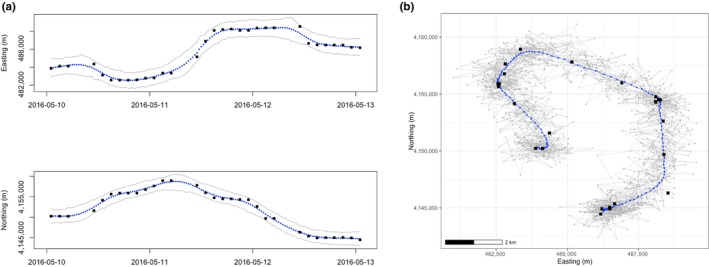
Locations of one lesser prairie‐chicken in one (a) and two (b) dimensional spaces over 3 days. Black squares represent recorded GPS data points, and blue circles represent the mean (expected value) of 1000 MCMC samples from the posterior distribution of the predicted location at Δ*t* = 0.5 h. (a) includes 95% credible intervals from the full MCMC sample and (b) includes 10 sampled trajectories in gray to visually represent the full MCMC sample.

A continuous trajectory of locations allows for inference about many attributes of movement, such as how long the animal spent in a place, when it interacted with a feature, or what behavior it may have exhibited (McClintock et al., 2014). However, current technology often limits transmitters to recording locations at interspersed and sometimes irregular intervals. Estimating the continuous trajectory of movement from these finite and irregularly spaced data points is the first step to answering applicable research questions. Continuous‐time animal movement models model an animal's coordinate location using a continuous function of time, allowing for the inference of positions over a fine‐time grid (Harris & Blackwell, 2013). This can be achieved by fitting a continuous‐time model for $y_{1,t}$ values (e.g., GPS transmitter recorded longitude or easting at time *t*) and fitting a separate continuous‐time model for $y_{2,t}$ values (e.g., GPS transmitter recorded latitude or northing at time *t*) and recombining them in two‐dimensional space (Figure 2). This separate modeling is based on assumed independence for the two movement directions, which is common in the literature (e.g., Chapter 6 in Hooten et al., 2017). Once fit, the model can estimate the animal's location $s_{t}$ at any time t, without requiring a fixed time step. These predicted values produce a continuous trajectory of the estimated $s_{t}$ locations that the animal traveled to.

Continuous‐time animal movement modeling is generally performed using some form of a GP model (Hooten et al., 2017). For example, the familiar continuous‐time correlated random walk model (Johnson et al., 2008) is a specifically formulated GP with a unique correlation function (Hooten & Johnson, 2017). GP models achieve highly accurate prediction of unknown values (e.g., an animal's location at any time) by estimating a flexible and nonlinear regression between data points. GPs are commonly applied to spatiotemporal data as they inherently account for the autocorrelation of input data within the model's covariance structure and utilize this autocorrelation to improve the accuracy of prediction (Gramacy, 2020).

### 2.3 Bayesian continuous‐time movement models

Utilizing a Bayesian GP movement model allows for quantification of the statistical uncertainty surrounding location estimates. Bayesian GP models are often applied to estimate continuous trajectories from telemetry data (Buderman et al., 2016; Hooten & Johnson, 2017; Johnson et al., 2011). As opposed to obtaining point estimates for GP parameters and associated location estimates of the trajectory, Bayesian movement models produce distributions of parameters and location estimates, thus providing quantification of the uncertainty surrounding trajectory estimation. This maintenance of statistical uncertainty measures throughout estimates is necessary for statistical comparison and inference.

### 2.4 Predicting trajectories

Within our framework, movement model parameters are not used directly for inference but instead as a means to estimate an animal's location at a given time. The model predicts the unrecorded locations between telemetry data points, thus estimating the animal's continuous trajectory. In the Bayesian formulation, these unrecorded locations are estimated by the posterior predictive distribution. Taking posterior predictive samples at small time intervals produces a distribution of expected trajectories at any desired resolution (e.g., predicted locations every hour, minute, second, etc.). For example, one may generate 1000 samples from the posterior predictive distribution of true location at a time point and use these samples to estimate the true location. Doing this at multiple (e.g., hourly) time points will estimate the predicted trajectory across time with associated uncertainty (Figure 2b).

The temporal resolution at which predictions are made is referenced as Δ*t*. The selection of Δ*t* depends on the temporal resolution of the data being analyzed and the scale of the desired inference. An appropriately small Δ*t* in relation to the movement phenomenon of interest (e.g., Δ*t* = 1 h and daily distance traveled is the goal of inference) allows for approximation to a continuous movement trajectory (Hanks et al., 2011). The selected value of Δ*t* presents a tradeoff between the accuracy of this approximation and computation time. The selection of Δ*t* must also consider the resolution of the available data (e.g., not predicting minute‐level locations from daily data). To avoid the requirement of an extremely fine resolution, as described by Noonan et al. (2019), we recommend only comparing trajectories and trajectory descriptors that have been approximated at equal Δ*t* values.

## 3 TREED GAUSSIAN PROCESSES

### 3.1 Nonstationarity

Stationary GPs fit a smooth function across time, meaning they assume the same parameterization throughout the data, and all data points affect model prediction and variance in all portions of the time series. Because of this assumption, stationary GPs do not perform well with large datasets or highly varied data (Gramacy & Lee, 2008). Animal movement data, even those describing a single trajectory, are usually large and highly varied. Animals rarely behave as a consistently parameterized function; rather, they perform variable movements at different periods of time. For example, a foraging movement will have a different parameterization than a traveling movement. Likewise, a resting movement will have lower variability in position than an active movement. This lack of a consistent pattern and parameterization over space or time within the data is termed nonstationarity.

When fitting a movement model to telemetry data, models must account for potential nonstationarity. If distinct animal movement states are modeled using a stationary process, uncertainty will be inflated unnecessarily, and movements of an animal over separate periods of time will inaccurately affect trajectory predictions (Gramacy, 2020). Different methods, such as temporal warping of GPs (Hooten & Johnson, 2017), nonstationary GP kernels (Torney et al., 2021), and time‐varying stochastic differential equations (Michelot et al., 2021), have been used to model the heterogeneity in telemetry data across smooth continuous‐time functions. However, animals do not always behave in smooth transitions of movement (e.g., sleeping to foraging, foraging to predator escape; Wolfson et al., 2022). Additionally, it is intuitive to model different animal movement patterns separately to prevent disparate movements from overly influencing predicted locations.

### 3.2 Treed Gaussian processes

Including a phenomenological treed partitioning component within the movement model can capture differing movement patterns and preserve the periodic abrupt transitions between them. Utilizing a machine learning model to perform this partitioning not only handles the nonstationarity of the movement trajectory but additionally leverages the high predictive accuracy provided by machine learning, aligning with our goal of trajectory interpolation (Figure 1; Wijeyakulasuriya et al., 2020). Because of their use of phenomenological treed partitioning, we recognize TGPs as a natural extension of the GP model for continuous‐time animal movement modeling. TGPs were developed by Gramacy (2005) by pairing stationary GPs and Bayesian treed partition models to better model nonstationary data and improve predictive accuracy. In the machine learning portion of the TGP model, a classification and regression tree is used to simultaneously partition the domain (time) into distinct regions and fit independent stationary GPs within those regions (e.g., at the nodes of the tree). This treed partitioning uses data values to make recursive splits based on rules that optimize classification and prediction. The classification tree is nested within a Bayesian partition model, meaning a distribution of tree structures is estimated and GP parameter estimates are conditional on the tree structure. As an additional benefit, partitioning increases computational efficiency from a stationary model because far smaller amounts of data are being fit to a GP at once. The Bayesian partition model is detailed in Gramacy and Lee (2008).

The use of partitioning allows for the fitting of a single treed model across the entire range of telemetry data. The TGP captures discrete shifts between movement patterns, similar to a hidden Markov model (Morales et al., 2004), but instead of modeling this process in discrete time, TGPs incorporate these jumps into the familiar continuous‐time GP model. While Wolfson et al. (2022) modeled discrete divides between continuous movement models using piecewise regression, the TGP model does not require the user to specify and join multiple models and instead utilizes the Bayesian classification tree to automatically fit independent GP models at the nodes of the tree. Thus, the TGP model retains the benefits of the GP model while increasing predictive accuracy and allowing for uncertainty estimates to better reflect the nonstationarity of the underlying process (Gramacy & Lee, 2008).

### 3.3 TGP implementation

Because TGPs are used across a diverse range of applications, a well‐developed R package exists for user‐friendly implementation of TGPs (Gramacy, 2007). Though this package was developed for general application and utilized by multiple fields, we offer its first application to animal movement modeling. Our demonstrated use of the tgp package allows for streamlined fitting of TGP models to animal telemetry data, and practitioners are not required to perform excessive manipulation of their data or write their own MCMC sampling algorithm. We outline steps for implementing the tgp package (version 2.4) in R to obtain inferences from GPS telemetry data for individual animals (see Appendix S2 for detailed steps and R code). Preprocessing of data is not required, though removal of data points arising from clear errors is expected prior to fitting the TGP model. Beyond these errors, there is no need to “thin” or remove any data points, nor must data fit a required frequency.

The TGP model is fit to easting and northing (or latitude and longitude) telemetry data separately. Bayesian TGP models are fit to these data using the btgpllm function in the tgp package. These fitted models are then used to make predictions within the desired time frame, with times of predictions depending on the practitioner selected Δ*t*. For the sequence of Δ*t* across the time period of inference, the tgp predict function is used to obtain samples from the posterior predictive distribution of the location at each Δ*t* time point. Minimal tuning is required for this sampling, but the selection of MCMC parameters is necessary (see Appendices S2 and S3 for more details). The resulting samples approximate the posterior predictive distribution of the continuous trajectories at Δ*t* resolution.

## 4 INFERENCE

### 4.1 Derived quantities

Once the trajectory for an individual animal has been estimated, statistics describing the trajectory can be used for inference and comparison (Johnson et al., 2011). Examples of statistics that summarize movement include distance traveled, turn angle, and the amount of time spent in a geographic area. These values are directly computed from a deterministic function of the model output (i.e., from the predicted trajectory). In the Bayesian formulation, such values are referenced as derived quantities. Bayesian derived quantities are treated as random variables and have their own posterior distribution and corresponding summary statistics (e.g., means and estimates of uncertainty such as credible intervals; Hobbs & Hooten, 2015). These uncertainty estimates are vital for comparing and drawing inferences from trajectories. Some common derived quantities of interest and their formulas are provided in Table 1. This is not an exhaustive list, but our outlined procedure follows for any deterministic function of the random variables (locations) and fixed variables (covariates). In addition to those listed in Table 1, investigators can easily perform transformations of derived quantities, which remain derived quantities. For example, cos(turn angle) and displacement × (time of day)^2^ have been computed for ecological reasons (Londe et al., 2022). Because any deterministic function can be used to compute a derived quantity from the predicted trajectory, the possibilities are infinite, and the desired movement descriptor must be carefully defined to address the specific research question.

**TABLE 1:** Four common derived quantities: displacement between times t1 and t2, turn angle at time t2 between times t1 and t3, residence time in area A over a time period T, and point of contact at time t.

Base derived quantity	MCMC formula	Extensions	Applications	Examples
Displacement	$d_{t1,t2}^{\left(k\right)}=\sqrt{\begin{array}{c} {\left(s_{1,t1}^{\left(k\right)}-s_{1,t2}^{\left(k\right)}\right)}^{2}+\\ {\left(s_{2,t1}^{\left(k\right)}-s_{2,t2}^{\left(k\right)}\right)}^{2}\; \end{array}}$	Average distance traveled over a period Speed Max speed over a period Average max speed over a period Max distance traveled Net displacement	Migration patterns Habitat quality Avoidance Behavior classification	Tanner et al. (2021) Buderman et al. (2018) Hanks et al. (2011)
Turn angle	$a_{t2}^{\left(k\right)}=\arccos\left(\frac{{\left(d_{t1,t2}^{\left(k\right)}\right)}^{2}+{\left(d_{t2,t3}^{\left(k\right)}\right)}^{2}-{\left(d_{t1,t3}^{\left(k\right)}\right)}^{2}\;}{2\;d_{t2,t3}^{\left(k\right)}d_{t1,t2}^{\left(k\right)}}\right)$	Average turn angle within a period Tortuosity Period or area where average turn angle crosses a threshold	Avoidance Behavior classification	Londe et al. (2022) Picardi et al. (2022) Postlethwaite et al. (2013)
Residence time	$r^{\left(k\right)}=\sum_{t=1}^{T}r_{t}^{\left(k\right)}$ $r_{t}^{\left(k\right)}=\left\{\begin{array}{c} \;1\;\text{if}\;s_{t}^{\left(k\right)}\in A\\ \;0\;\text{if}\;s_{t}^{\left(k\right)}\notin A\; \end{array}\;\right.$ where A is the area of interest	Proportion of time in habitat type	Habitat and resource selection Home range determination	Brown et al. (2017) Pozdnyakov et al. (2014) Buderman et al. (2018)
Point of contact	$s_{t}^{\left(k\right)}$ such that: $s_{t}^{\left(k\right)}=p$ where p is the point of interest	Probability of contact	Road crossings Encountering conspecifics Human–wildlife conflict Disease spread	Long et al. (2022) Eriksen et al. (2009) Dodge et al. (2021)

*Note*: Formulas for computation are based on $s_{t}^{\left(k\right)}$, the *k*th MCMC sample of the animal's location at time t. Examples are provided of possible extensions and applications of these base derived quantities, as well as examples of their uses in the literature. Examples are non‐exhaustive, and many more applications exist.

### 4.2 Derived quantity estimation

After obtaining samples from the trajectory's distribution, sampling from the posterior distribution of the derived quantity is as simple as implementing the deterministic function across the trajectory samples. Because the derived quantity is a function of the trajectory random variable, the equivariance property of MCMC ensures that the derived quantity is also a random variable and that the transformed MCMC sample is a sample from the distribution of that derived quantity (Hobbs & Hooten, 2015).

We iterate through the *K* MCMC samples of predicted trajectories, transforming each *k*th sample from the posterior predictive distribution into a sample from the chosen derived quantity. This applies to any derived quantity formula (Table 1). We demonstrate the MCMC approximation for the example derived quantity of displacement at each Δ*t*. From the *K* samples of predicted trajectories S at Δ*t* resolution, we individually transform each *k* sampled trajectory into a sequence of the derived quantity at Δ*t* resolution. For this example, this is as simple as iteratively computing the distance between two points:

$$d_{t1,t2}^{\left(k\right)}=\sqrt{{\left(s_{1,t1}^{\left(k\right)}-s_{1,t2}^{\left(k\right)}\right)}^{2}+{\left(s_{2,t1}^{\left(k\right)}-s_{2,t2}^{\left(k\right)}\right)}^{2}\;}$$

Performing the derived quantity calculation (in this case, the Euclidian distance formula) at the Δ*t* interval and across all *K* samples produces *K* samples of derived quantities at Δ*t* resolution. Detailed code for a displacement example is provided in Appendix S2. Formulas for sampling other derived quantities are in Table 1. Appendix S1 provides additional information on applying the Monte Carlo approximation to the underlying continuous‐time model.

### 4.3 Transforming temporal scale

The output of the derived quantity computation is samples of the derived quantity at every Δ*t* interval for the entire time period. For example, this could be 744 hourly displacements over a month across 1000 samples. This distribution of temporally fine‐scale derived quantities is not immediately interpretable and must be summarized at a scale that can be used for inference. The flexibility of our modeling framework allows transformations (e.g., averages) of the desired derived quantity to be easily computed at any scale greater than the initially set Δ*t*.

Though it may be tempting to reduce the large, fine‐scale derived quantity distribution into summary statistics and transform these, it is necessary to transform the entire distribution to preserve its properties (e.g., uncertainty measures). To continue with the displacement example, we can transform the distribution of 744 hourly displacements into a distribution of the average daily distance traveled in that month. First, we sum within days within each trajectory sample to obtain 1000 samples each with 31 daily totals. The 31 daily total distances are then averaged within each of the 1000 trajectory samples. This transformation maps a distribution of displacements at a fine Δ*t* to a distribution of a single value. The distribution of this desired value (average daily distance traveled in the month) can be summarized for interpretation using statistics such as its mean and 95% credible interval. This example is shown visually in Appendix S4. The code for performing derived quantity transformations is detailed in Appendix S2.

### 4.4 Population‐level inference

Population‐level inference is made by aggregating individual‐level derived quantities, and thus is itself a derived quantity (Buderman et al., 2016). Instead of being a function of one random variable (one trajectory), population‐level statistics are a function of multiple random variables (multiple trajectories). The equivariance property of the population‐level derived quantity will hold when MCMC samples are aligned properly (see Chapter 8.3 in Hobbs & Hooten, 2015; Appendix S4). This fits within our MCMC framework for sampling from the posterior distribution of the movement trajectories and their resulting derived quantities. Following the previous example, we first obtain *N* individual distributions of the average daily distance traveled in the month for *N* different animals. These *N* distributions are aggregated into a single distribution of the average daily distance traveled in the month for the population of *N* individuals. In the MCMC framework, this aggregation is accomplished by averaging across all individuals within each of the *K* samples (Hobbs & Hooten, 2015). The code for this sampling is provided in Appendix S2. A visualization of this example is provided in Appendix S4.

## 5 CASE STUDY: LESSER PRAIRIE‐CHICKEN MOVEMENT UNDER DIFFERING LAND USE

### 5.1 Background

We utilized a GPS telemetry dataset collected from female lesser prairie‐chickens to present two illustrative applications of our approach. Because lesser prairie‐chickens are a species of conservation concern, researchers collected these data to assess movement patterns and habitat selection (Gulick, 2019; Lautenbach et al., 2021; Verheijen et al., 2021). Comparing movements across habitats and management types can answer novel research questions for this species and help guide management. Our first illustration demonstrates how the TGP model captures unusual movements, while our second illustration applies the inferential framework to compare movement across habitats. For both illustrations, we fit TGP models and sampled from posterior predictive distributions for locations at Δ*t* = 1 h across the study period. We used the base tgp MCMC settings for fitting models and a MCMC sample size of 1000 (burn‐in of 2000, total MCMC iterations of 12,000, and thinned by 10) for the posterior predictive distribution of movement trajectories. We confirmed that these settings achieved convergence by visually evaluating trace plots and ensuring that the effective sample size for a predicted location was 1000. Appendix S2 provides a step‐by‐step code tutorial with practice data. Due to the listing of lesser prairie‐chicken populations as threatened or endangered under the United States Endangered Species Act, data and code for the full case study analyses may be provided with reasonable request.

### 5.2 Illustration 1: Modeling unusual movements

**FIGURE 3:**
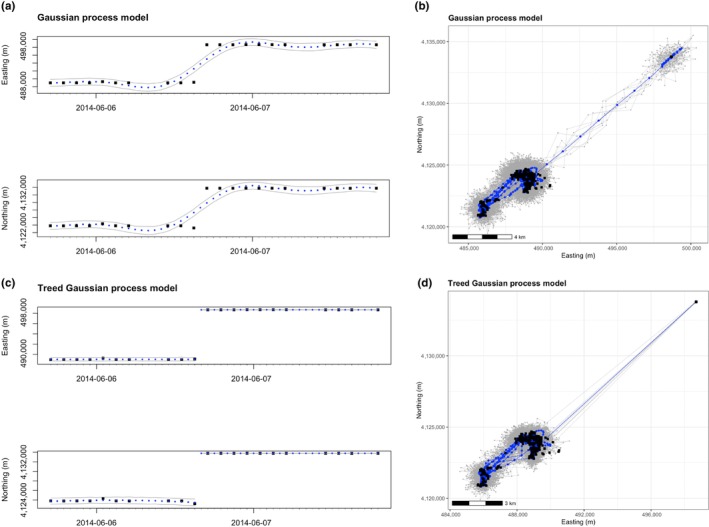
Results for Illustration 1 in one (a, c) and two (b, d) dimensions. Results from both stationary Gaussian process modeling (a, b) and treed Gaussian process modeling (c, d) are presented. Black squares represent recorded GPS data points, and blue circles represent the mean (expected value) of 1000 MCMC samples from the posterior distribution of the predicted location at Δ*t* = 0.5 h. (a) and (c) include 95% credible intervals from the full MCMC samples. (b) and (d) include 10 sampled trajectories in gray to visually represent the full MCMC sample. (a) and (c) show data across 2 days, while (b) and (d) show data across 2 months.

First, we demonstrate the ability of the TGP model to handle extreme or unusual animal movements. We identified one such movement when an individual lesser prairie‐chicken traveled at least 14.2 km in the 2 h between GPS recordings. Because an abrupt movement of this magnitude is not uncommon for a female lesser prairie‐chicken and multiple GPS fixes were recorded, these data points were determined to be produced by movement and not GPS error. We fit separate TGP and GP models to this individual and compared the resulting treed and non‐treed trajectory distributions in both one and two dimensions (Figure 3). The treed model more closely follows the data during the extreme movement, while the non‐treed model smooths over the notable movement present in the data. In attempting to fit two drastically different movement patterns with one continuous function, the smoothed model skews the underlying movement pattern provided by the data and fails to detect the notable movement of 14 km in 2 h (Figure 3a). Additionally, the GP results in increased variance and lack of fit within the two sections (visible in the wider 95% credible intervals of the GP model, Figure 3). Treed partitioning gracefully handles these modeling challenges with a more precise fit to these data, demonstrating the superior ability of TGPs for modeling real animal movements that fail to behave in a predictable manner.

### 5.3 Illustration 2: Ecological inference

**FIGURE 4:**
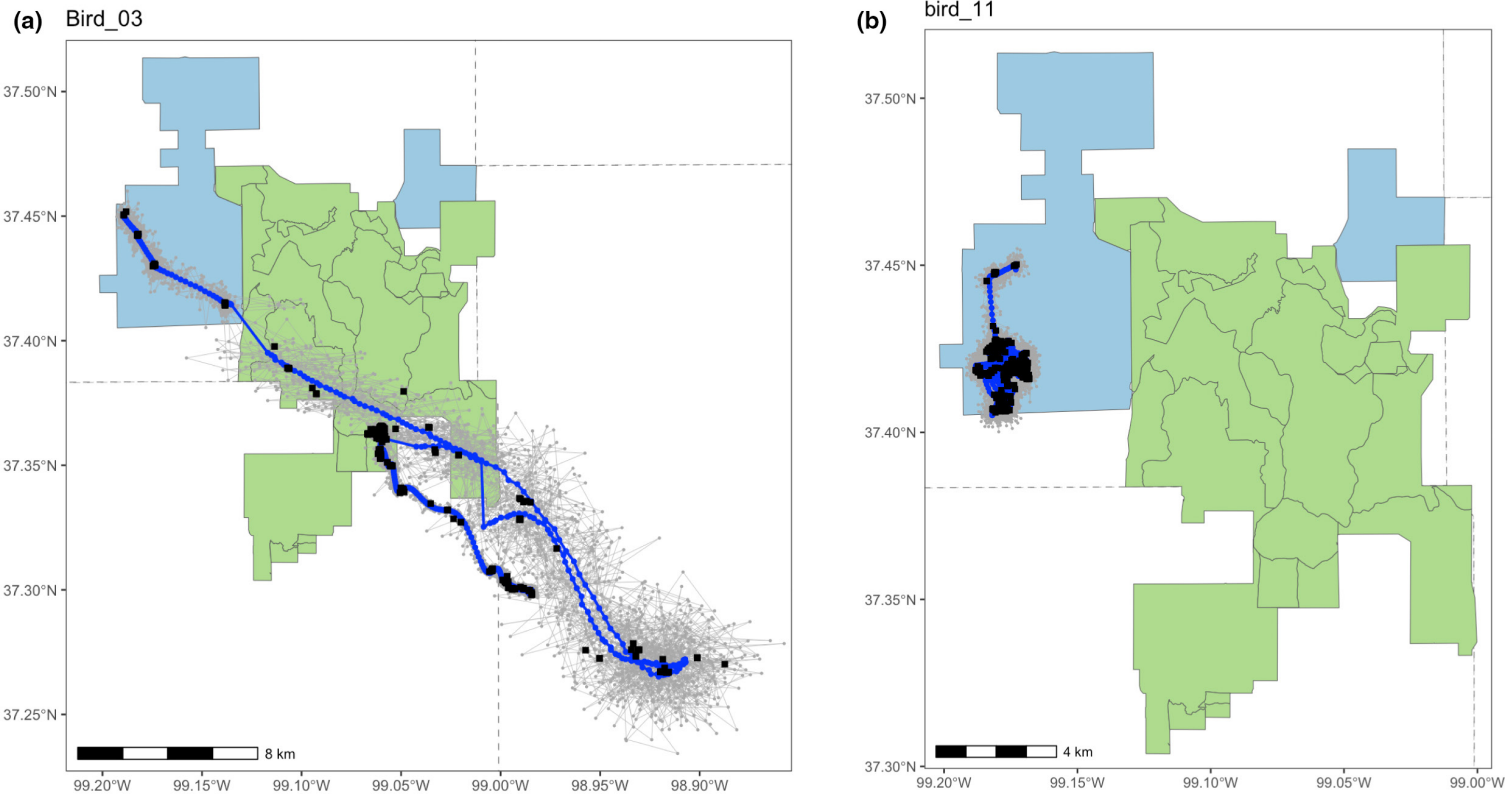
Locations of two lesser prairie‐chickens (a, b) over 2 months in relation to different cattle grazing systems. Rotationally grazed land is shown in blue, and patch‐burn grazed land is shown in green. Black squares represent recorded GPS data points, and blue circles represent the mean (expected value) of 1000 MCMC samples from the posterior distribution of the predicted location at Δ*t* = 1 h. Ten sample trajectories from the full MCMC sample are represented in gray.

**FIGURE 5:**
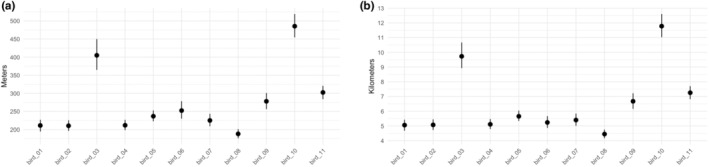
Individual average hourly displacements (a) and average daily distances traveled (b) for the 11 female lesser prairie‐chickens. For each individual bird, points represent the mean value of 1000 MCMC samples from the posterior distribution of the derived quantity, and error bars show 95% credible intervals. Values are the average across the season of the total distance traveled in each hour (a) or each day (b), at Δ*t* = 1 h.

Our second illustration demonstrates application of the TGP model and example inference using one season of lesser prairie‐chicken data (2016‐04‐15 through 2016‐06‐15). For the 11 individual birds within this sub‐dataset, we sampled from four derived quantities that describe differences in movements observed in the individual lesser prairie‐chickens (Figure 4). First, we sampled from the posterior distribution of hourly displacements across the study period for each bird. We then transformed these into samples of average hourly displacement across the whole season for each bird (Figure 5a). To represent this on a different temporal scale, we transformed hourly displacements into the daily total distance traveled for each bird (Figure 5b). We observed individual‐level differences in average displacements, demonstrated by the lack of overlap of the two derived quantities' 95% Bayesian credible intervals across birds.

**FIGURE 6:**
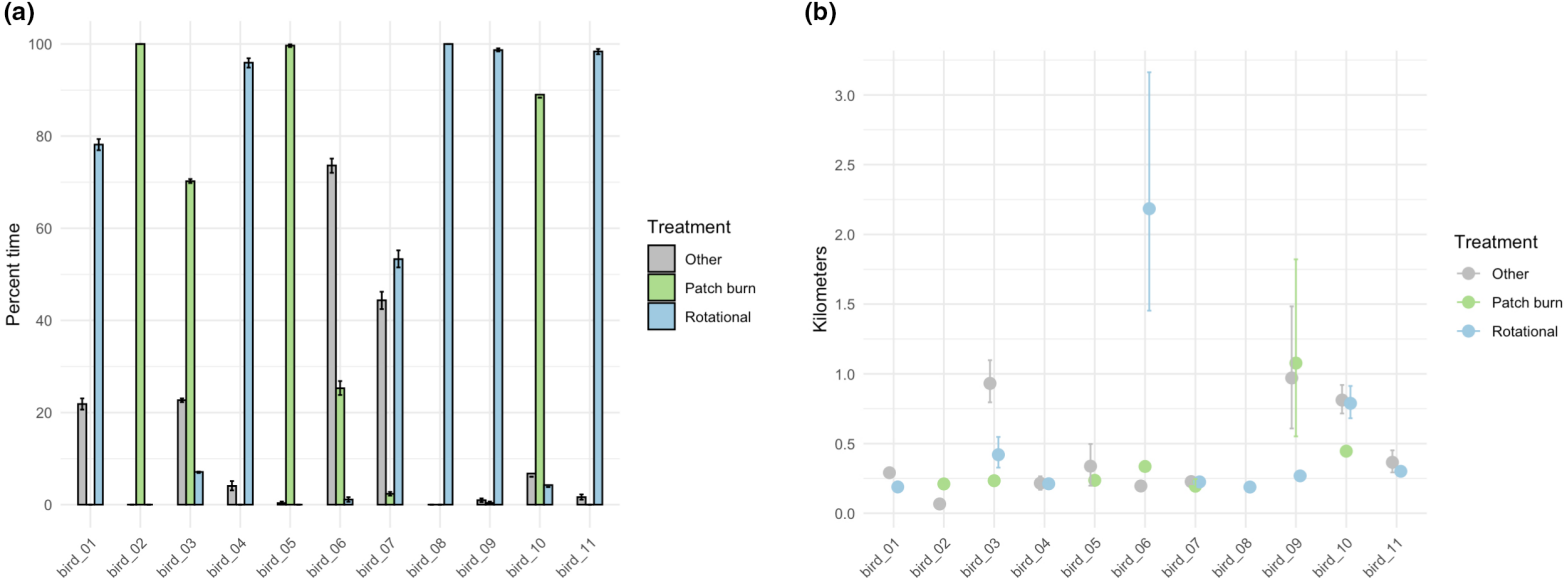
Treatment‐based derived quantities for the 11 individual female lesser prairie‐chickens. Percent time spent by the individual bird in each grazing treatment is shown in (a) and average hourly displacement by the individual bird while within each grazing treatment is shown in (b). Point estimates represent the mean of the 1000 MCMC samples from the posterior distribution of the derived quantity, and error bars represent the 95% credible intervals.

**FIGURE 7:**
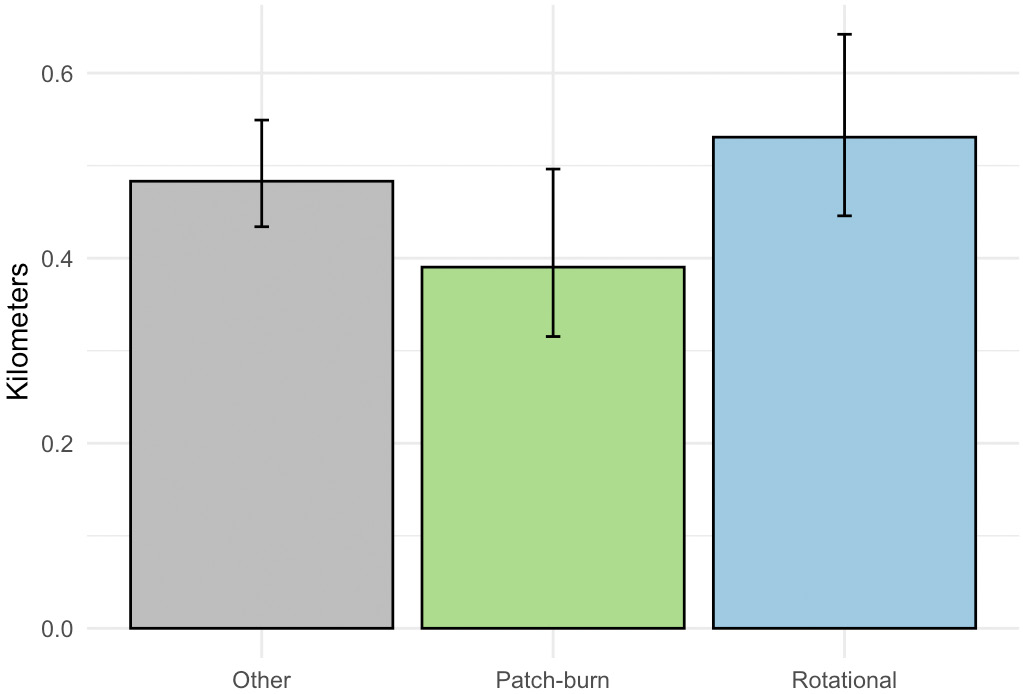
Population‐level average hourly displacements within each grazing treatment for the 11 female lesser prairie‐chickens. Point estimates represent the means of the aggregated posterior distributions of the derived quantity for each treatment, and error bars represent 95% Bayesian credible intervals.

We then integrated spatial data on ecologically relevant grazing classifications into our analysis to demonstrate possible management applications (see Gulick, 2019 for additional information). At each Δ*t* point in the sample of 1000 trajectories, we classified the location of each bird as within one of three mutually exclusive grazing treatments. By working within the MCMC framework, we obtained point estimates of the percent of time spent in each treatment for each bird, as well as associated 95% Bayesian credible intervals (Figure 6a). Continuing with these classified samples, we sampled hourly displacements within each of the three grazing treatments. We transformed these sampled displacements to average hourly displacements within each grazing treatment, revealing six birds where sampled distributions of movement across grazing treatments did not have overlapping 95% Bayesian credible intervals (Figure 6b). We then computed the aggregated population‐level‐derived quantity of average hourly displacement within each grazing treatment, averaged across the 11 birds (Figure 7). The accompanying Bayesian 95% credible intervals show clear overlap for the movement metric of interest across grazing treatments, effectively reducing a complex dataset with multiple individuals and thousands of telemetry points to a set of easily interpretable values.

## 6 DISCUSSION

The distinct partitions of TGP models reflect how animals change their movement in abrupt ways (e.g., flushing, ambushing prey, reacting to a conspecific). The ability to model these extreme movements and rare events is valuable to wildlife ecologists, as unusual movements such as traveling to a water source during a drought, evading a predator, or sheltering during extreme weather may be a determining factor of survival for an individual. Incorporating these movements into a model could greatly benefit understanding a species' resource requirements.

In addition to modeling the intrinsic nonstationarity of animal telemetry data, the use of machine learning within the TGP model increases the ease and automation of animal movement modeling. Utilizing a prespecified, phenomenological model increases the automation of trajectory estimation, freeing researchers from the burden of developing custom movement models and allowing for a shift in focus from model development and parameter interpretation to prediction and derived quantity estimation. The existence of an established R package for TGP implementation greatly increases the accessibility of this already simplified modeling, and our short Appendix S2 facilitates the application of TGPs to telemetry data using R. Aside from the removal of clear telemetry errors, there is no required preprocessing or discarding of data, and the core modeling component is only a few lines of code. This allows practitioners to estimate movement descriptors and answer applied questions from their telemetry data without extensive programming or experience with MCMC algorithms. Fitting TGP models within the tgp package is relatively fast (within the order of minutes or hours, depending on the study period), and additional optional modifications to achieve faster speeds are described in Appendix S2. Because TGPs are used across multiple fields, increases in computational speed made elsewhere can benefit wildlife movement modeling (Gramacy, 2020).

In addition to providing accurate prediction and accessible implementation, the TGP model's Bayesian formulation allows for statistical inference and application to research questions. The five derived quantities estimated in Illustration 2 compress complex individual and population movement trajectories into statistically comparable values and demonstrate the flexibility of our framework for both the inclusion of covariates and different scales of analysis. This flexibility allows for creativity in developing derived quantities that suit the research question of interest. Utilizing different derived values to describe movement is common in both Bayesian and frequentist inference, and there is a wide body of work on this topic and its applications (Table 1). Our modeling framework provides trajectories that directly and easily estimate a distribution for any derived quantity of interest and thus is an ideal starting point for continued development and applications of quantitative descriptors of animal movement.

Derived quantities that describe multiple trajectories (e.g., multiple individuals within a population) are worth emphasizing because of their value to practitioners. While population‐level movement modeling is an area of developing research (e.g., Hooten et al., 2016), our current modeling framework can perform population‐level inference by fitting multiple individual movement models and then estimating derived quantities at the population level. This ability to compare movements of populations of individuals across treatments holds great potential for wildlife management and ecology applications.

Understanding how animals move within their environments is key to data‐driven wildlife management. Though researchers have access to large amounts of telemetry data, current continuous‐time modeling methods can either fail to model nonstationarity in movement data or require advanced model‐building experience to implement. The TGP model provides a machine‐learning‐based interpolation of movement trajectories that incorporates changes in movement pattern and is conducive to flexible inference without requiring the user to custom specify a movement model. From these estimated trajectories, our framework allows researchers to derive statistically comparable descriptors of individual or group movement. The use of TGP modeling within our inferential framework facilitates a shift in animal movement modeling from focusing on model construction and interpretation to focusing instead on the inference that can be made from easy‐to‐implement continuous‐time movement models.

## AUTHOR CONTRIBUTIONS

**Camille J. Rieber:** Conceptualization (equal); formal analysis (equal); methodology (equal); writing – original draft (lead); writing – review and editing (equal). **Trevor J. Hefley:** Conceptualization (equal); methodology (equal); writing – review and editing (equal). **David A. Haukos:** Conceptualization (equal); data curation (lead); methodology (equal); writing – review and editing (equal).

## CONFLICT OF INTEREST STATEMENT

The authors declare no conflicts of interest.

## Supporting information


Appendix S1

Appendix S2

Appendix S3

Appendix S4

## Data Availability

A portion of the data can be accessed within the Appendix S2 R script file. This data excerpt allows for a demonstration of coding methods within the Appendix S2 tutorial. The lesser prairie‐chicken location data used for the case study were recorded on private land and represent locations of a threatened and endangered species under the United States Endangered Species Act. Therefore, these spatially explicit data are sensitive and cannot be transformed for public access. Locations may be provided by the authors following an appropriate request.

## References

[ece311447-bib-0001] Brown, L. M. , Fuda, R. K. , Schtickzelle, N. , Coffman, H. , Jost, A. , Kazberouk, A. , Kemper, E. , Sass, E. , & Crone, E. E. (2017). Using animal movement behavior to categorize land cover and predict consequences for connectivity and patch residence times. Landscape Ecology, 32(8), 1657–1670. 10.1007/s10980-017-0533-8

[ece311447-bib-0002] Buderman, F. E. , Hooten, M. B. , Ivan, J. S. , & Shenk, T. M. (2016). A functional model for characterizing long‐distance movement behaviour. Methods in Ecology and Evolution, 7(3), 264–273. 10.1111/2041-210X.12465

[ece311447-bib-0003] Buderman, F. E. , Hooten, M. B. , Ivan, J. S. , & Shenk, T. M. (2018). Large‐scale movement behavior in a reintroduced predator population. Ecography, 41(1), 126–139. 10.1111/ecog.03030

[ece311447-bib-0004] Dodge, S. , Su, R. , Johnson, J. , Simcharoen, A. , Goulias, K. , Smith, J. L. , & Ahearn, S. C. (2021). ORTEGA: An object‐oriented time‐geographic analytical approach to trace space‐time contact patterns in movement data. Computers, Environment and Urban Systems, 88, 101630. 10.1016/j.compenvurbsys.2021.101630

[ece311447-bib-0005] Eriksen, A. , Wabakken, P. , Zimmermann, B. , Andreassen, H. P. , Arnemo, J. M. , Gundersen, H. , Milner, J. M. , Liberg, O. , Linnell, J. , Pedersen, H. , Sand, H. , Solberg, E. , & Storaas, T. (2009). Encounter frequencies between GPS‐collared wolves (*Canis lupus*) and moose (*Alces alces*) in a Scandinavian wolf territory. Ecological Research, 24(3), 547–557. 10.1007/s11284-008-0525-x

[ece311447-bib-0006] Glennie, R. , Adam, T. , Leos‐Barajas, V. , Michelot, T. , Photopoulou, T. , & McClintock, B. T. (2023). Hidden Markov models: Pitfalls and opportunities in ecology. Methods in Ecology and Evolution, 14, 43–56. 10.1111/2041-210X.13801

[ece311447-bib-0007] Gramacy, R. B. (2005). *Bayesian treed Gaussian process models* [Doctoral dissertation, University of California, Santa Cruz].

[ece311447-bib-0008] Gramacy, R. B. (2007). tgp: An R package for Bayesian nonstationary, semiparametric nonlinear regression and design by treed Gaussian process models. Journal of Statistical Software, 19, 1–46. 10.18637/jss.v019.i09 21494410

[ece311447-bib-0009] Gramacy, R. B. (2020). Surrogates: Gaussian process modeling, design, and optimization for the applied sciences. Chapman and Hall/CRC. 10.1201/9780367815493

[ece311447-bib-0010] Gramacy, R. B. , & Lee, H. K. H. (2008). Bayesian treed Gaussian process models with an application to computer modeling. Journal of the American Statistical Association, 103(483), 1119–1130. 10.1198/016214508000000689

[ece311447-bib-0011] Gulick, C. K. (2019). *Effects of working grassland management on lesser prairie‐chicken resource selection within home ranges and during dispersal events* [Master's thesis, Kansas State University].

[ece311447-bib-0012] Hanks, E. M. , Hooten, M. B. , Johnson, D. S. , & Sterling, J. T. (2011). Velocity‐based movement modeling for individual and population level inference. PLoS One, 6(8), e22795. 10.1371/journal.pone.0022795 21931584 PMC3154913

[ece311447-bib-0013] Harris, K. J. , & Blackwell, P. G. (2013). Flexible continuous‐time modeling for heterogeneous animal movement. Ecological Modeling, 255, 29–37. 10.1016/j.ecolmodel.2013.01.020

[ece311447-bib-0014] Hobbs, N. T. , & Hooten, M. B. (2015). Bayesian models. Princeton University Press. 10.23943/princeton/9780691159287.003.0005

[ece311447-bib-0015] Hooten, M. B. , Buderman, F. E. , Brost, B. M. , Hanks, E. M. , & Ivan, J. S. (2016). Hierarchical animal movement models for population‐level inference. Environmetrics, 27(6), 322–333. 10.1002/env.2402

[ece311447-bib-0016] Hooten, M. B. , & Johnson, D. S. (2017). Basis function models for animal movement. Journal of the American Statistical Association, 112(518), 578–589. 10.1080/01621459.2016.1246250

[ece311447-bib-0017] Hooten, M. B. , Johnson, D. S. , McClintock, B. T. , & Morales, J. M. (2017). Animal movement: Statistical models for telemetry data. CRC Press.

[ece311447-bib-0018] Johnson, D. S. , London, J. M. , & Kuhn, C. E. (2011). Bayesian inference for animal space use and other movement metrics. Journal of Agricultural, Biological, and Environmental Statistics, 16, 357–370.

[ece311447-bib-0036] Johnson, D. S. , London, J. M. , Lea, M.‐A. , & Durban, J. W. (2008). Continuous‐time correlated random walk model for animal telemetry data. Ecology, 89(5), 1208–1215. 10.1890/07-1032.1 18543615

[ece311447-bib-0019] Lautenbach, J. D. , Haukos, D. A. , Lautenbach, J. M. , & Hagen, C. A. (2021). Ecological disturbance through patch‐burn grazing influences lesser prairie‐chicken space use. The Journal of Wildlife Management, 85(8), 1699–1710. 10.1002/jwmg.22118

[ece311447-bib-0020] Londe, D. W. , Elmore, R. D. , Davis, C. A. , Hovick, T. J. , Fuhlendorf, S. D. , & Rutledge, J. (2022). Why did the chicken not cross the road? Anthropogenic development influences the movement of a grassland bird. Ecological Applications, 32(3), e2543. 10.1002/eap.2543 35080784

[ece311447-bib-0021] Long, J. A. , Webb, S. L. , Harju, S. M. , & Gee, K. L. (2022). Analyzing contacts and behavior from high frequency tracking data using the wildlifeDI R package. Geographical Analysis, 54(3), 648–663. 10.1111/gean.12303

[ece311447-bib-0022] McClintock, B. T. , Johnson, D. S. , Hooten, M. B. , Ver Hoef, J. M. , & Morales, J. M. (2014). When to be discrete: The importance of time formulation in understanding animal movement. Movement Ecology, 2(1), 1–14. 10.1186/S50462-014-0021-6 25709830 PMC4337762

[ece311447-bib-0023] Michelot, T. , Glennie, R. , Harris, C. , & Thomas, L. (2021). Varying‐coefficient stochastic differential equations with applications in ecology. Journal of Agricultural, Biological and Environmental Statistics, 26, 446–463. 10.1007/s13253-021-00450-6

[ece311447-bib-0024] Morales, J. M. , Haydon, D. T. , Frair, J. , Holsinger, K. E. , & Fryxell, J. M. (2004). Extracting more out of relocation data: Building movement models as mixtures of random walks. Ecology, 85(9), 2436–2445. 10.1890/03-0269

[ece311447-bib-0025] Noonan, M. J. , Fleming, C. H. , Akre, T. S. , Drescher‐Lehman, J. , Gurarie, E. , Harrison, A. L. , Kays, R. , & Calabrese, J. M. (2019). Scale‐insensitive estimation of speed and distance traveled from animal tracking data. Movement Ecology, 7(1), 1–15. 10.1186/s40462-019-0177-1 31788314 PMC6858693

[ece311447-bib-0026] Patterson, T. A. , Parton, A. , Langrock, R. , Blackwell, P. G. , Thomas, L. , & King, R. (2017). Statistical modeling of individual animal movement: An overview of key methods and a discussion of practical challenges. AStA Advances in Statistical Analysis, 101, 399–438. 10.1007/s10182-017-0302-7

[ece311447-bib-0027] Picardi, S. , Coates, P. , Kolar, J. , O'Neil, S. , Mathews, S. , & Dahlgren, D. (2022). Behavioural state‐dependent habitat selection and implications for animal translocations. Journal of Applied Ecology, 59(2), 624–635. 10.1111/1365-2664.14080

[ece311447-bib-0028] Postlethwaite, C. M. , Brown, P. , & Dennis, T. E. (2013). A new multi‐scale measure for analysing animal movement data. Journal of Theoretical Biology, 317, 175–185. 10.1016/j.jtbi.2012.10.007 23079283

[ece311447-bib-0029] Pozdnyakov, V. , Meyer, T. , Wang, Y. B. , & Yan, J. (2014). On modeling animal movements using Brownian motion with measurement error. Ecology, 95(2), 247–253. 10.1890/13-0532.1 24669719

[ece311447-bib-0030] Tanner, E. P. , Tanner, A. M. , Fuhlendorf, S. D. , Elmore, R. D. , Davis, C. A. , & Polo, J. A. (2021). Land enrolled in the Conservation Reserve Program supports roosting ecology of the lesser prairie‐chicken. Global Ecology and Conservation, 32, e01916. 10.1016/j.gecco.2021.e01916

[ece311447-bib-0031] Torney, C. J. , Morales, J. M. , & Husmeier, D. (2021). A hierarchical machine learning framework for the analysis of large scale animal movement data. Movement Ecology, 9, 1–11. 10.1186/s40462-021-00242-0 33602302 PMC7893961

[ece311447-bib-0032] Tuia, D. , Kellenberger, B. , Beery, S. , Costelloe, B. R. , Zuffi, S. , Risse, B. , Mathis, A. , Mathis, M. W. , Langevelde, F. , Burghardt, T. , Kays, R. , Klinck, H. , Wikelski, M. , Couzin, I. D. , van Horn, G. , Crofoot, M. C. , Stewart, C. V. , & Berger‐Wolf, T. (2022). Perspectives in machine learning for wildlife conservation. Nature Communications, 13(1), 792. 10.1038/S51467-022-27980-y PMC8828720 35140206

[ece311447-bib-0033] Verheijen, B. H. , Plumb, R. T. , Gulick, C. K. , Hagen, C. A. , Robinson, S. G. , Sullins, D. S. , & Haukos, D. A. (2021). Breeding season space use by lesser prairie‐chickens (*Tympanuchus pallidicinctus*) varies among ecoregions and breeding stages. The American Midland Naturalist, 185(2), 149–174. 10.1674/0003-0031-185.2.149

[ece311447-bib-0034] Wijeyakulasuriya, D. A. , Eisenhauer, E. W. , Shaby, B. A. , & Hanks, E. M. (2020). Machine learning for modeling animal movement. PLoS One, 15(7), e0235750. 10.1371/journal.pone.0235750 32716917 PMC7384613

[ece311447-bib-0035] Wolfson, D. W. , Andersen, D. E. , & Fieberg, J. R. (2022). Using piecewise regression to identify biological phenomena in biotelemetry datasets. Journal of Animal Ecology, 91(9), 1755–1769. 10.1111/1365-2656.13779 35852382 PMC9540865

