# Supplementary material for: Treed Gaussian processes for animal movement modeling
Source: Ecol Evol. 2024 Jun 2;14(6):e11447. doi: 10.1002/ece3.11447 (PMC11144715; doi:10.1002/ece3.11447)
Supplement: Supplementary file 1 — Appendix S1 [file ECE3-14-e11447-s002.pdf]

Appendix S1: Movement model notation and specification

In this appendix, we provide details on the notation and models for the application of treed Gaussian processes to animal movement. This guide is not necessary for applying treed Gaussian processes but provides deeper understanding of the statistical underpinning of our model.

Terms:

$\mathbf{y}_t \equiv (y_{1,t}, y_{2,t})$	One recorded animal location at time *t*
$\mathbf{Y} \equiv \begin{bmatrix} y_{1,1} & y_{2,1} \\ \vdots & \vdots \\ y_{1,n} & y_{2,n} \end{bmatrix}$	n x 2 matrix of all recorded locations
$\mathbf{s}_t \equiv (s_{1,t}, s_{2,t})$	One true animal location at time *t*
$\mathbf{S} \equiv \begin{bmatrix} s_{1,1} & s_{2,1} \\ \vdots & \vdots \\ s_{1,m} & s_{2,m} \end{bmatrix}$	m x 2 matrix of true animal locations at temporal resolution of Δt
$f(\mathbf{S})$	Deterministic function of locations

Models:

$\mathbf{Y} \sim [\,\mathbf{Y} \mid \mathbf{S}, \sigma^2\,]$	Data model
σ^2	Parameter for data location error
$\mathbf{S} \sim [\,\mathbf{S} \mid \boldsymbol{\theta}\,]$	Process model

S is distributed as a treed Gaussian process with the parameters $\boldsymbol{\theta}$.

Parameter models are pre-specified within the `tgp` function in program R.

Posterior Distribution for Discrete Approximation of True Trajectory:

$$[\mathbf{S} \mid \mathbf{Y}] = \int [\mathbf{S} \mid \boldsymbol{\theta}]\,[\boldsymbol{\theta} \mid \mathbf{Y}]\,d\boldsymbol{\theta}$$

Expected Value of a Derived Quantity of the Trajectory:

$$E(f(\mathbf{S}) \mid \mathbf{Y}) = \int f(\mathbf{S})\,[\mathbf{S} \mid \boldsymbol{\theta}]\,[\boldsymbol{\theta} \mid \mathbf{Y}]\,d\boldsymbol{\theta}$$

Monte Carlo Approximation of Expected Value of a Derived Quantity:

$$E(f(\mathbf{S}) \mid \mathbf{Y}) \approx \sum_{k=1}^{K} \frac{f(\mathbf{S}^{(k)})}{K}$$

The following table outlines the relationships between the underlying theoretical continuous time movement model, the practical computer implemented discrete approximation of the continuous time model (described on the previous page), and the Monte Carlo sampling-based algorithm used to fit this model to our data and estimate derived quantities.

**Continuous Time Model**	**Discrete Approximation**	**Monte Carlo Approximation**
Posterior predictive distribution of continuous stochastic process:<br>$\mathbf{s}(t) \sim [\,\mathbf{s}(t) \mid \mathbf{Y}\,]$<br>**s** is defined as the true trajectory, modelled as a treed Gaussian process. **s**(*t*) is a continuous function of continuous time.	Posterior predictive distribution of discretized continuous stochastic process:<br>$\mathbf{S} \sim [\,\mathbf{S} \mid \mathbf{Y}\,]$<br>**S** is a discretization of the true continuous trajectory. **S** is a finite vector of true locations at each Δt time.	One trajectory sampled from the posterior distribution of the discretized continuous stochastic process:<br>$\mathbf{S}^{(k)} \sim [\,\mathbf{S} \mid \boldsymbol{\theta}^{(k)}\,]$<br>To obtain a sample of size K from the posterior predictive distribution of $[\,\mathbf{S} \mid \mathbf{Y}\,]$:<br>1. Take a draw of $\boldsymbol{\theta}^{(k)}$ from $[\boldsymbol{\theta} \mid \mathbf{Y}]$<br>2. Take a draw of $\mathbf{S}^{(k)}$ from $[\,\mathbf{S} \mid \boldsymbol{\theta}^{(k)}\,]$<br>3. Repeat K times
<u>Apply derived quantity function</u><br>$f(\mathbf{s}(t))$<br>Ex. instantaneous velocity:<br>$v(t) \sim \mathbf{s}'(t)$<br>*Result: distribution of function providing velocity across continuous time*	<u>Apply derived quantity function</u><br>$f(\mathbf{S})$<br>Ex. average velocity across interval (*t1, t2*):<br>$v_{t1,t2} = \sqrt{\frac{(s_{1,t1} - s_{1,t2})^2 + (s_{2,t1} - s_{2,t2})^2}{t2 - t1}}$<br>*Result: distribution of m-1 dimension vectors V of average velocities at each time t*	<u>Apply derived quantity function</u><br>$f(\mathbf{S}^{(k)})$<br>Ex. average velocity across interval (*t1, t2*):<br>$v^{(k)}_{t1,t2} = \sqrt{\frac{(s^{(k)}_{1,t1} - s^{(k)}_{1,t2})^2 + (s^{(k)}_{2,t1} - s^{(k)}_{2,t2})^2}{t2 - t1}}$<br>*Result: K samples of m-1 dimension vectors* $\boldsymbol{V}^{(k)}$ *of average velocities at each time t*
<u>Transform derived quantity</u><br>$g(f(\mathbf{s}(t)))$<br>Ex. average daily distance travelled over a period of *a* days:<br>$\sim \frac{1}{a}\int_0^T v(t)dt$<br>*Result: distribution of a scalar value*	<u>Transform derived quantity</u><br>$g(f(\mathbf{S}))$<br>Ex. average daily distance travelled over a period of *a* days:<br>$\dot{\sim} \frac{1}{a}\sum_{t=1}^{m} v_{t,t+1}$<br>*Result: distribution of a scalar value*	<u>Transform derived quantity</u><br>$g(f(\mathbf{S}^{(k)}))$<br>Ex. average daily distance travelled over a period of *a* days:<br>$\approx \frac{1}{a}\sum_{t=1}^{m} v^{(k)}_{t,t+1}$<br>*Result: sample of size K of a scalar value*
