# Supplementary material for: Treed Gaussian processes for animal movement modeling
Source: Ecol Evol. 2024 Jun 2;14(6):e11447. doi: 10.1002/ece3.11447 (PMC11144715; doi:10.1002/ece3.11447)
Supplement: Supplementary file 2 — Appendix S2 [file ECE3-14-e11447-s003.pdf]

Appendix S2: tutorial to apply TGP modeling to telemetry data

This R Markdown document provides code for fitting Bayesian treed Gaussian process models to animal telemetry data and estimating derived quantities describing movement. This appendix demonstrates application to one individual animal, and must be repeated for multiple individuals or separate treatments.

To work through this appendix, we use 100 consecutive GPS points from one lesser prairie-chicken. After using this example to follow the code it can be replaced with your own data and research questions/derived quantities.

We rely on Robert Gramacy's `tgp` package (version 2.4) to fit movement models.

```
#install.package("tgp")
library(tgp)
```

Step 1) Read in data

The dataframe we use requires **X** (GPS easting or longitude), **Y** (GPS northing or latitude), and **t.obs** (GPS recorded time) columns, which may require some manipulation of your GPS data. Data should already be cleaned and checked for location error outliers, as described in Appendix S3 section 3.

Modeling is performed either using lat-long or UTM coordinates; predicted trajectories will be outputted in whichever form the coordinates are read in as. This means the user may decide which coordinate form to use. If modeling in lat-long, trajectories will have to be converted to UTM to perform derived quantity computations such as distance between points. However, UTM may pose problems for large datasets that span multiple zones. The lesser prairie-chicken dataset utilizes UTM.

Pre-formatted example toy lesser prairie-chicken data can be read in the .Rmd document:

```
individual<- cbind(timestamp,individual)
names(individual) <- c("timestamp","X", "Y")
head(individual)
```

```
##             timestamp        X       Y
## 1 2018-04-15 00:00:00 495544.2 4137085
## 2 2018-04-15 02:00:00 495544.2 4137066
## 3 2018-04-15 04:00:00 495529.2 4137066
## 4 2018-04-15 06:00:00 495544.2 4137066
## 5 2018-04-15 08:00:00 495514.1 4137085
## 6 2018-04-15 10:00:00 495514.2 4137122
```

Add t.obs column:

t.obs provides a standardized time measure for the model to intake. Units will depend on your time scale, but counting in hours is common. Hour "0" can relate to any time within or outside the dataset, and won't affect the modeling, as you'll transform back to the natural time scale for your results.

We used t.obs = hours since first recorded GPS point.

```
#convert to hours since start
individual$t.obs <- (as.numeric(individual$timestamp- min(individual$timestamp)))/3600
```

Step 2) Fit TGP Model

Fit the model to the X (easting) and Y (northing) coordinates separately

Bayesian treed Gaussian process with jumps to the limiting linear model (within the `tgp` package) Note that for large datasets and without any parallelization this may be a time consuming step.

Inclusion of jumps to the limiting linear model in the TGP model allows the model to fit a linear model in partitions where the data do not require a Gaussian process, see Gramacy & Lee (2008) for more information.

```
model_x <- btgpllm(X=individual$t.obs, #fit time as explanatory variable
                   Z= individual$X, #fit easting location as dependent variable
                   bprior="b0", #set uninformative hierarchical normal prior
                   verb=0, #don't print progress meter. Set =1 to print progress meter.
                   pred.n = F) #prevents prediction at t.obs points for faster model
                               #fitting (all prediction will be made in later step)

 #same as above settings, but fit to northing location:
model_y <- btgpllm(X=individual$t.obs,
                   Z= individual$Y,
                   bprior="b0",
                   verb=0,
                   pred.n = F)
```

Step 3) Predict paths

Set MCMC hyperparameters

Prediction under the treed Gaussian Process requires MCMC sampling. In the `tgp` package this requires the tuning parameter of BTE = c(burn in, total samples, thinning value). Setting of these MCMC sampling parameters is explained in Appendix S3 section 2, and we recommend an understanding of MCMC sampling at the level of Hobbs and Hooten (2015) or Hooten and Hefley (2019).

We recommend prototyping your data with small BTE settings first, for example `c(400,500,2)`. Once you have prototyped and code is running without error, increase the BTE and MCMC sample size. `bte <- c(2000, 12000, 10)` is a good starting place for an appropriately large sample size, but this selection is discussed further in Appendix S3 section 2. Evaluation of the MCMC sample is provided at the end of this section.

```
bte <- c(2000,12000,10) #EDIT this line to prototype
MCMC_sample_size <- (bte[2] - bte[1])/bte[3] #do not edit this line
```

Set Δt

Choice of Δt is described in depth in Appendix S3 section 1. A Δt of 1 hour is reasonable for many applications.

```
delta.t <-  1 # 1 hour. This may be edited.
```

Use fitted model to predict

This chunk does not require editing. It will sample from the posterior predictive distribution of locations at each Δt time point.

Note that for large datasets and without any parallelization this may be a time consuming step and should be prototyped on a small dataset and small BTE first.

```r
season.end <- max(individual$t.obs)
XX <- seq(0, season.end, by = delta.t) #points we want prediction at

#### build storage table
all_paths <-  data.frame(matrix(ncol = 1+ 2*MCMC_sample_size, nrow = length(XX)) )
names(all_paths)[1] <- "t"
all_paths$t <- XX
seq.x <- seq(2,ncol(all_paths), by = 2) #sequence of even numbers
names(all_paths)[seq.x] <- paste0('x', 1:MCMC_sample_size)
seq.y <- seq(3,ncol(all_paths), by = 2) #sequence of odd numbers not 1
names(all_paths)[seq.y] <- paste0('y', 1:MCMC_sample_size)

#### tgp model fitting transformation
# The tgp model fitting utilizes a specific transformation of the data used to fit
#the model. This ensures that prespecified priors, the scale hyperparameter, etc.
#set within the package work for all types of data. The specific transformation
#used by the package is to transform data that have a mean of 0 and range of 1.

## make your transformation constants from the data once:
z.x <-individual$X
z.x.ranged <- -0.5 +(z.x - min(z.x))/(max(z.x) - min(z.x))
z.y <-individual$Y
z.y.ranged <- -0.5 +(z.y - min(z.y))/(max(z.y) - min(z.y))

#### compute predictions
predicted_x <- predict(model_x, XX, pred.n=FALSE,  BTE=bte,  MAP=FALSE, trace=T)
posterior_predictives_x_ <- predicted_x$trace$preds$ZZ
predicted_y <-  predict(model_y, XX, pred.n=FALSE, BTE=bte, MAP=FALSE, trace=T)
posterior_predictives_y <- predicted_y$trace$preds$ZZ
#Setting MAP = FALSE when making prediction means that the MCMC sampling will start at
#the MAP (maximum a posteriori probability) tree, but then continue in Bayesian form
#and sample across the full posterior distribution of trees. This means different MCMC
#samples will often be from different possible trees (different partitioning solutions).

# Back transform to undue model fitting scaling:
posterior_predictives_x <- t(posterior_predictives_x_ + mean(z.x.ranged) + 0.5)*
  (max(z.x) - min(z.x)) + min(z.x);
posterior_predictives_y <- t(posterior_predictives_y + mean(z.y.ranged) + 0.5)*
  (max(z.y) - min(z.y)) + min(z.y);
# take transpose of matrix so it's set up with times are rows and samples are columns
#instead of vice versa

### seperate these into the table
all_paths[,seq.x] <- posterior_predictives_x ; #fill in all the xs
all_paths[,seq.y] <- posterior_predictives_y #fill in all the ys
```

The `all_paths` data frame holds samples (across columns) of X and Y trajectory locations over each Δt interval (across rows). This is a large table of dimension: T x (MCMC sample size * 2 + 1). Where T = (end time - start time)/delta_t

```r
dim(all_paths)
```

```
## [1]  229 2001
```

```r
all_paths[1:5,1:7]
```

```
##   t       x1      y1       x2      y2       x3      y3
## 1 0 495528.5 4137101 495498.6 4137129 495508.4 4137026
## 2 1 495517.9 4137052 495581.7 4137157 495538.9 4137061
## 3 2 495508.8 4137055 495528.8 4137060 495517.5 4137031
## 4 3 495536.5 4136956 495481.3 4137049 495550.2 4137126
## 5 4 495475.2 4137065 495548.0 4137086 495561.8 4137102
```

After `all_paths` is outputted, this would be a good time to export `all_paths` and save it on your device if your dataset is large.

Though it may be tempting to elimininate this storage required for the `all_paths` data frame by predicting the paths and computing the derived quantities simultaneously, saving the full MCMC sampled distribution of fine-resolution trajectories allows the flexibility to change scale or derived quantities as research questions change or are added. Because predicting these full trajectories is the most time consuming step, we recommend saving the full predicted trajectory sample outside of R for future use.

To check the effective sample size of your MCMC samples, run the following code after you've sampled from the posterior predictive distribution. A large effective sample size (close to the MCMC sample size) implies good mixing of the sample and low autocorrelation. Low effective sample size suggests autocorrelation that may require increased thinning (increased E in BTE settings).

```r
#install.packages("coda")
library(coda)
sample <- as.numeric(posterior_predictives_x_[,1])
effectiveSize(sample)
```

```
## var1
## 1000
```

To examine a trace plot of your MCMC samples, run the following after you've sampled from the posterior predictive distribution.

```r
plot(1:MCMC_sample_size, sample,type="l")
```

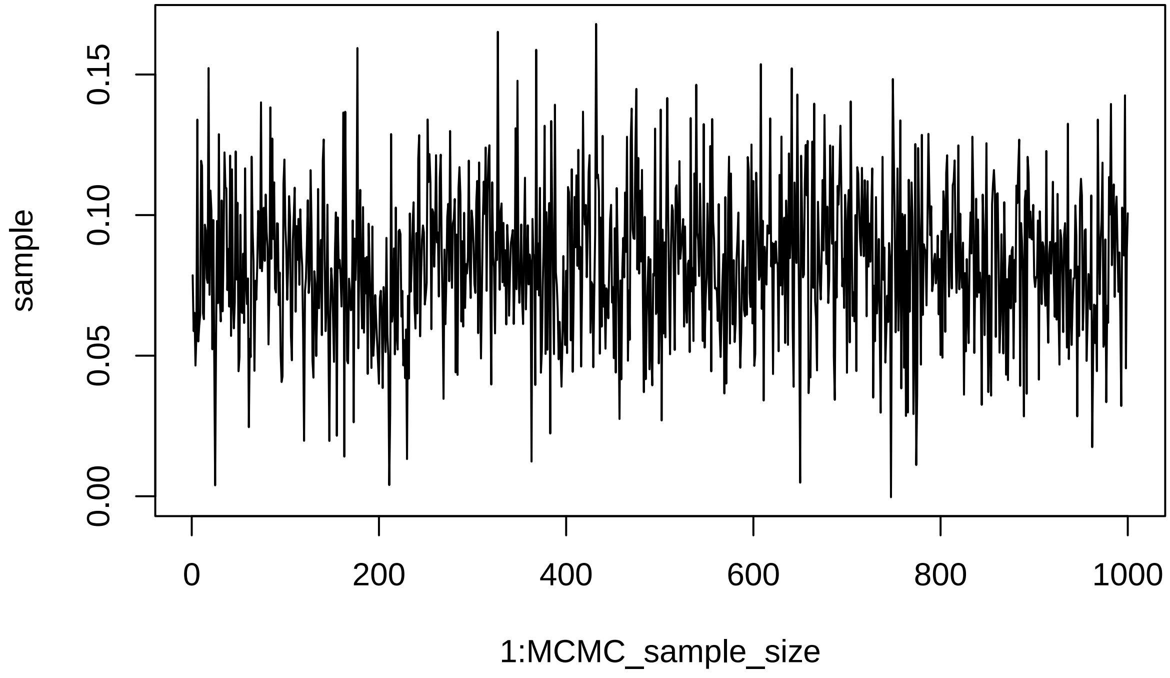

Plot predicted trajectory

We can visualize the predicted trajectory in both one and two dimensions. Black squares plot recorded GPS data points and blue circles plot the mean (expected value) of the MCMC samples from the posterior distribution of predicted locations at the set Δt inteval. Plotting code can be found in the Appendix S2.Rmd file

One dimension, including 95% credible intervals (gray lines) from the full MCMC sample:

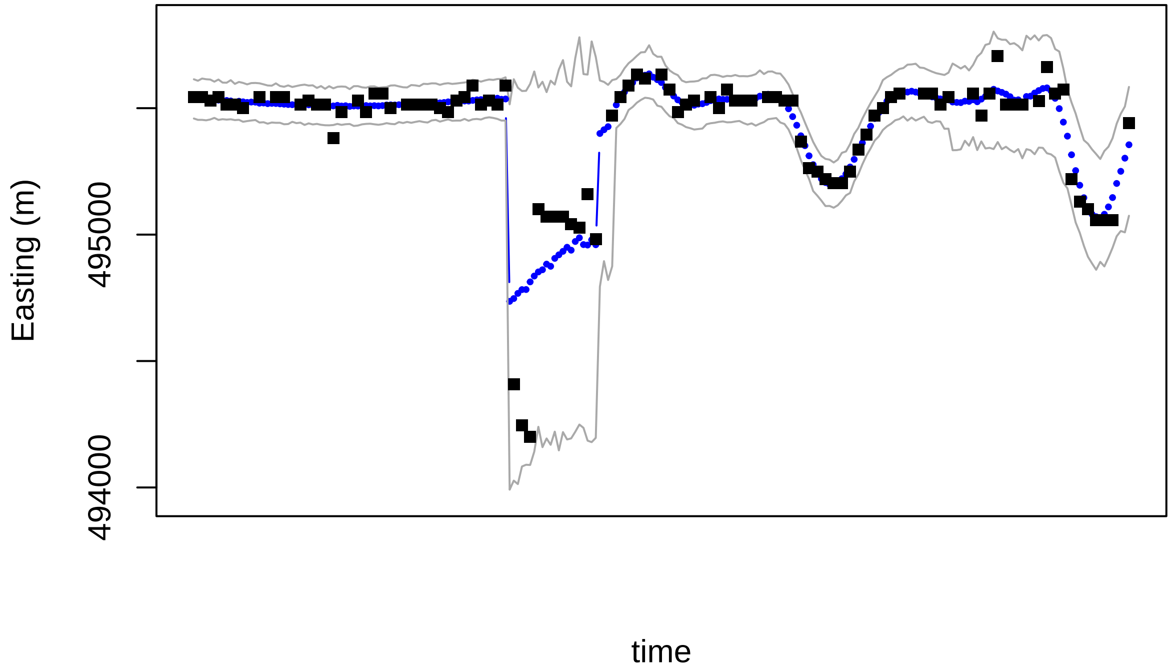

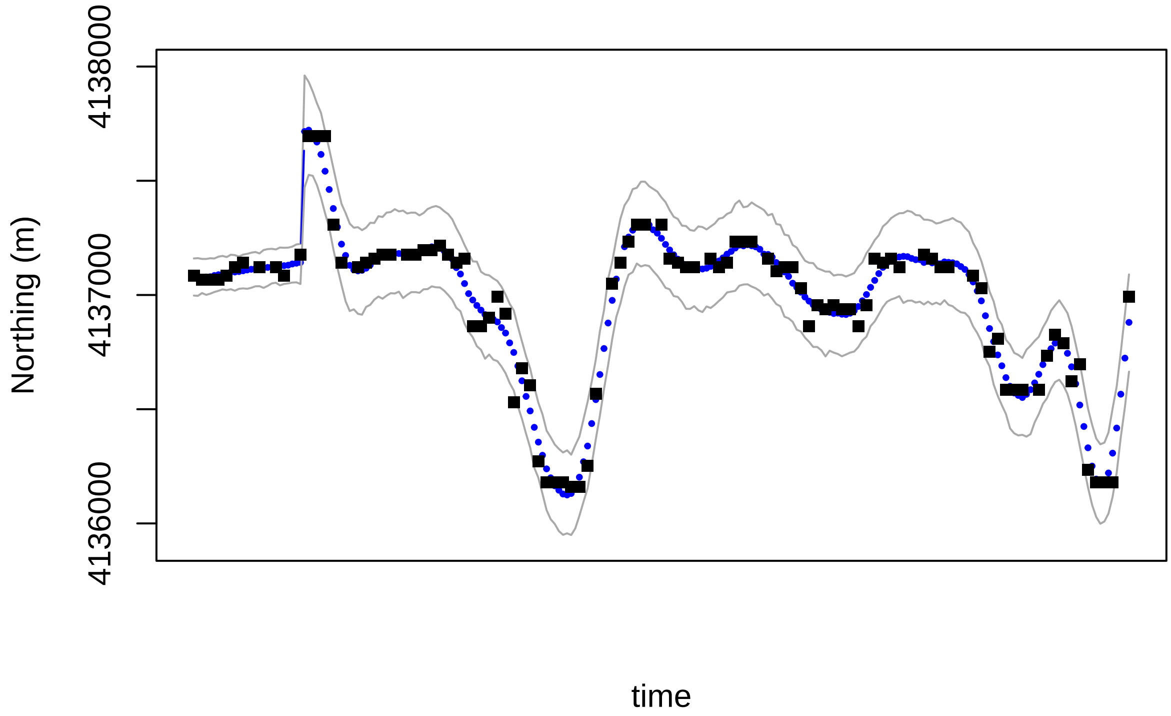

Two dimensions, including 20 sampled trajectories in gray to visually represent the MCMC sample:

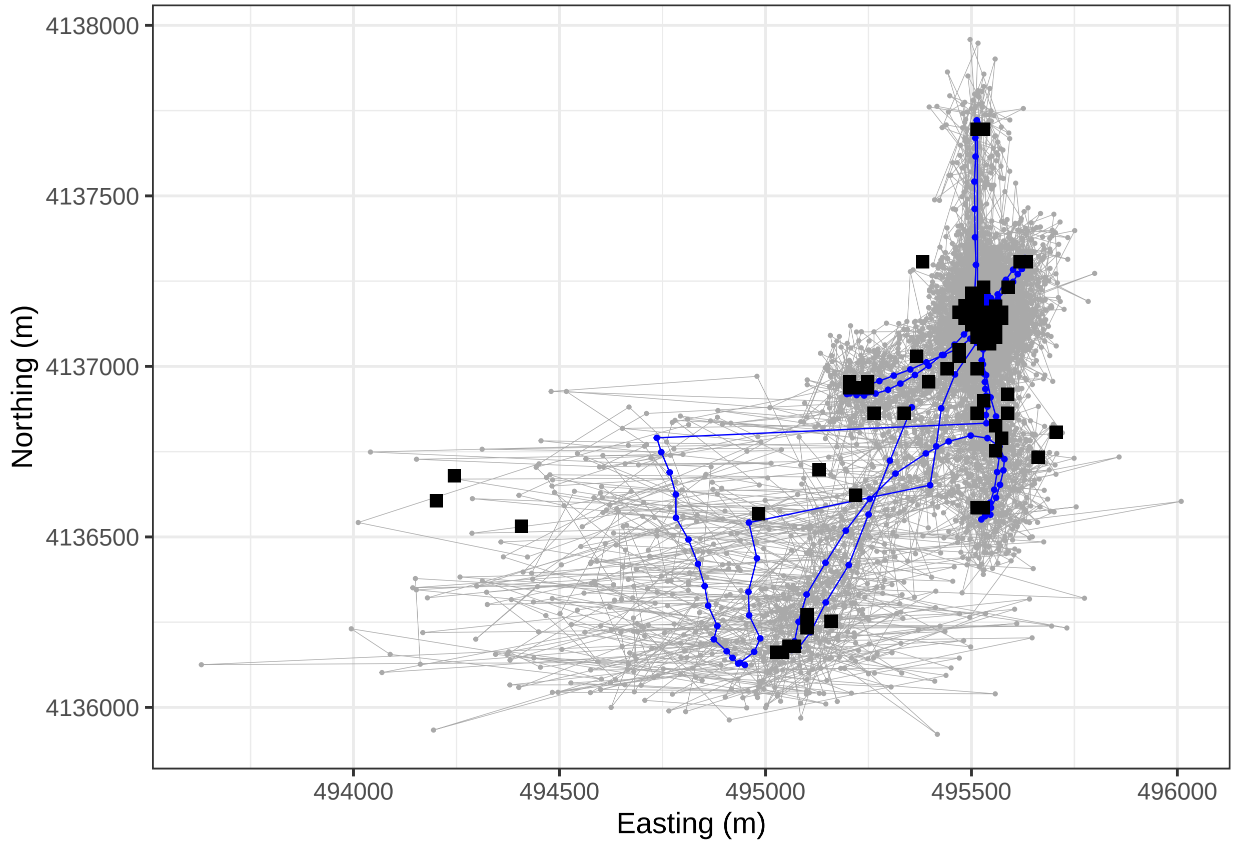

Step 4) Compute derived quantity at Δt resolution

First we use the `all_paths` table to compute the derived quantity at the scale of Δt (e.g., hourly). This is the scale that `all_paths` is already at. Beginning at this resolution allows us to then transform to any larger desired scale (e.g., daily, weekly).

It may be helpful to follow along with the displacement example in Appendix S4 as well, and we've noted how each code step aligns with each step in Appendix S4.

Your derived quantity

You will have to edit this section depending on your derived quantity. As the options for derived quantities are infinite, this may take some creativity. Refer to Table 1 of the manuscript for basic derived quantity functions that can be built off of.

You will have to write your derived quantity function here:

The displacement function used in section 4.2 of the manuscript is provided as an example.

```
my_DQ_fun <- function(t){
  #a function of one delta t instant, represented by t (the row in all_paths)
  return(sqrt((all_paths[t,colStart ] - all_paths[t+1, colStart])^2  +
                (all_paths[t, colStart+1 ] - all_paths[t+1,colStart+1])^2  ))
}
# this is the distance formula calling (x1,y1) and (x2,y2) from the all_paths data
#frame, indexed at time t
```

The following step takes a sample from the distribution of the derived quantity at each Δt time point (in this

case hourly displacement), producing a table of dimension: (time points - 1) x (MCMC samples + 1)

[arrow 1 in Appendix S4]

```
#create storage table for the DQ:
all_DQs <-  data.frame(matrix(ncol = 1+ MCMC_sample_size, nrow = length(XX)-1) )
names(all_DQs)[1] <- "t"
all_DQs$t <- XX[-1]
names(all_DQs)[-1] <- paste0('d', 1:MCMC_sample_size)

for(j in 1:(ncol(all_DQs)-1) ){  #loop through all the MCMC samples
  colStart <- 2*j
  #one sample of DQs:
  for(i in 1:nrow(all_DQs)){  # loop through all the times (fill out one column)
    DQ <- my_DQ_fun(i)
    all_DQs[i, j+1] <- DQ
  }
}
dim(all_DQs)
```

```
## [1]  228 1001
```

```
all_DQs[1:5,1:7]
```

```
##   t         d1        d2        d3       d4        d5        d6
## 1 1  50.157555  87.59828  46.56989 86.26498  76.59762  17.09818
## 2 2   9.698462 110.13612  36.83727 85.78532  82.99768 117.17840
## 3 3 102.612293  48.84976 100.21151 56.03458  30.06817  77.15624
## 4 4 124.852649  76.67394  26.46007 77.09990 135.93751  84.27752
## 5 5 130.059189  82.43286  69.23130 83.98338 120.74447  30.03611
```

We now have a data frame of samples of the derived quantity (in this case hourly displacement) at each Δt point, with d1-d1000 indexing the 1000 samples.

Step 5) Output inference at desired scale

The above 252,000 samples of different hourly displacements may not be at the temporal scale of interest, so we can transform it into our desired value: average daily total distance traveled over the study period.

Though it may be tempting to just average all values in the table, we must maintain alignment of MCMC samples (see Chapter 8.3 in Hobbs & Hooten, 2015). See Appendix S4 for further walking through of this example.

These steps will vary based on your transformations and desired ending derived quantity

In our example, if we want to transform to **daily total distance traveled averaged across the season**, we first take daily sums within each of the MCMC samples (columns) separately.

[arrow 2 in Appendix S4]

We used the dplyr and lubridate packages to make this easier.

```
#All derived quantities values are in t.obs, and we need to transform them back to natural
#time to get dailies:

library(lubridate) #Installing the package lubridate is useful for this.

# we will use the original timestamps now so that we can get dailies:
time_start <- min(individual$timestamp) + 60*60  #adding one hour since this was hour 0
```

```r
#and displacement starts at hour 1
time_end <- time_start  + (max(all_DQs$t)-1)*60*60  #adding all the hours in the DQ
#predictions to get end time

time <- seq(time_start , time_end  , by = 'hours')

daily <- cbind(time, all_DQs)
day <- floor_date(daily$time, "day") # get the day of each date
daily <- cbind(day, all_DQs)

library(dplyr)
daily_DQs <- daily %>%
  group_by(day) %>%
  summarise(across(d1:d1000, sum))

dim(daily_DQs)
```

```
## [1]   10 1001
```

```r
daily_DQs[1:5,1:7]
```

```
## # A tibble: 5 x 7
##   day                    d1    d2     d3     d4    d5    d6
##   <dttm>              <dbl> <dbl>  <dbl>  <dbl> <dbl> <dbl>
## 1 2018-04-15 00:00:00 1701. 1751.  1464.  2123. 2135. 1360.
## 2 2018-04-16 00:00:00 3917. 3937.  4060.  3367. 2486. 3866.
## 3 2018-04-17 00:00:00 2662. 3035.  1859.  2679. 2526. 2820.
## 4 2018-04-18 00:00:00 6077. 8347. 12637. 13747. 9926. 9169.
## 5 2018-04-19 00:00:00 4851. 4981.  6060. 11631. 4872. 4838.
```

This gives a distribution (1000 samples) of daily total distance traveled.

To get 1000 samples of daily distance traveled averaged across the season, we take column (sample) averages.

[arrow 3 in Appendix S4]

```r
average_daily_DQs <- as.vector(colMeans(daily_DQs[,-1]))
length(average_daily_DQs)
```

```
## [1] 1000
```

```r
hist(average_daily_DQs, breaks = 30, xlab = "Average daily distance traveled (meters)",
     main = "Samples from posterior distribution")
```

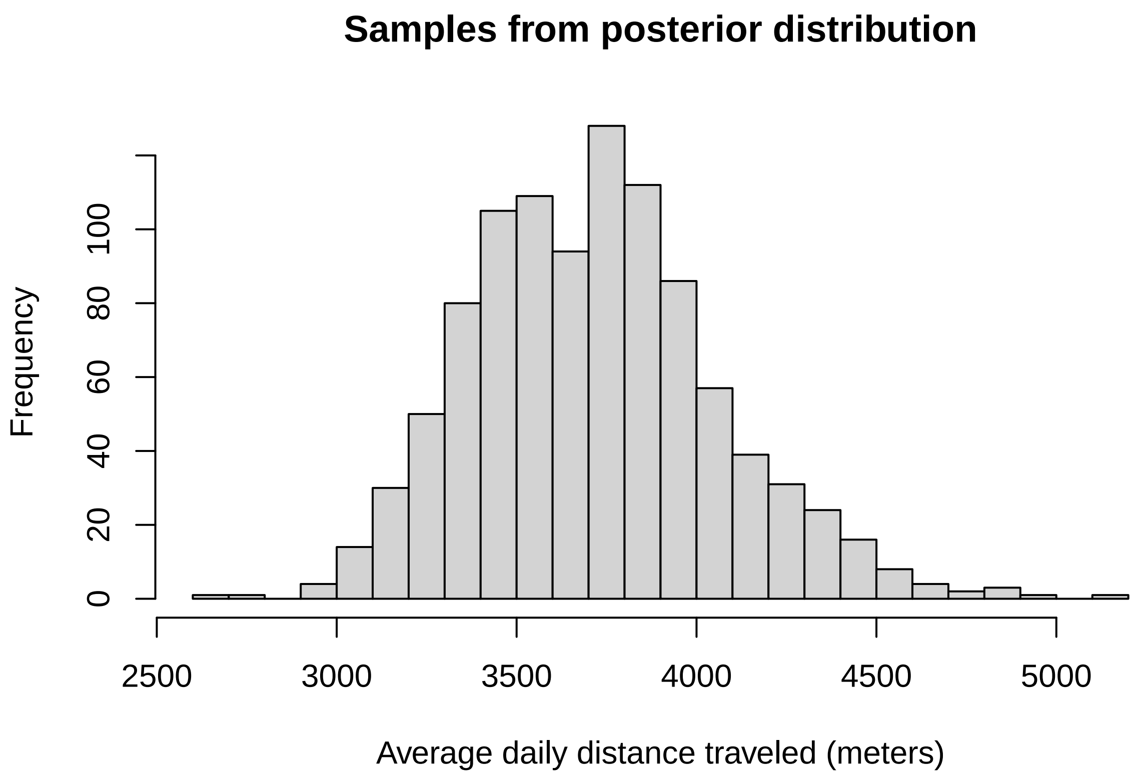

We can summarize this sample of average daily distance traveled using the mean and 95% credible region:

```
mean(average_daily_DQs)
```

```
## [1] 3718.514
```

```
c(quantile(average_daily_DQs, 0.025), quantile(average_daily_DQs, 0.975))
```

```
##     2.5%    97.5%
## 3115.746 4460.501
```

```
hist(average_daily_DQs, breaks = 30, xlab = "Average daily distance traveled (meters)",
     main = "Samples from posterior distribution")
abline(v = c( mean(average_daily_DQs), quantile(average_daily_DQs, 0.025),
              quantile(average_daily_DQs, 0.975)), col = c("red", "blue", "blue"),
              lty=c(1,2,2) )
```

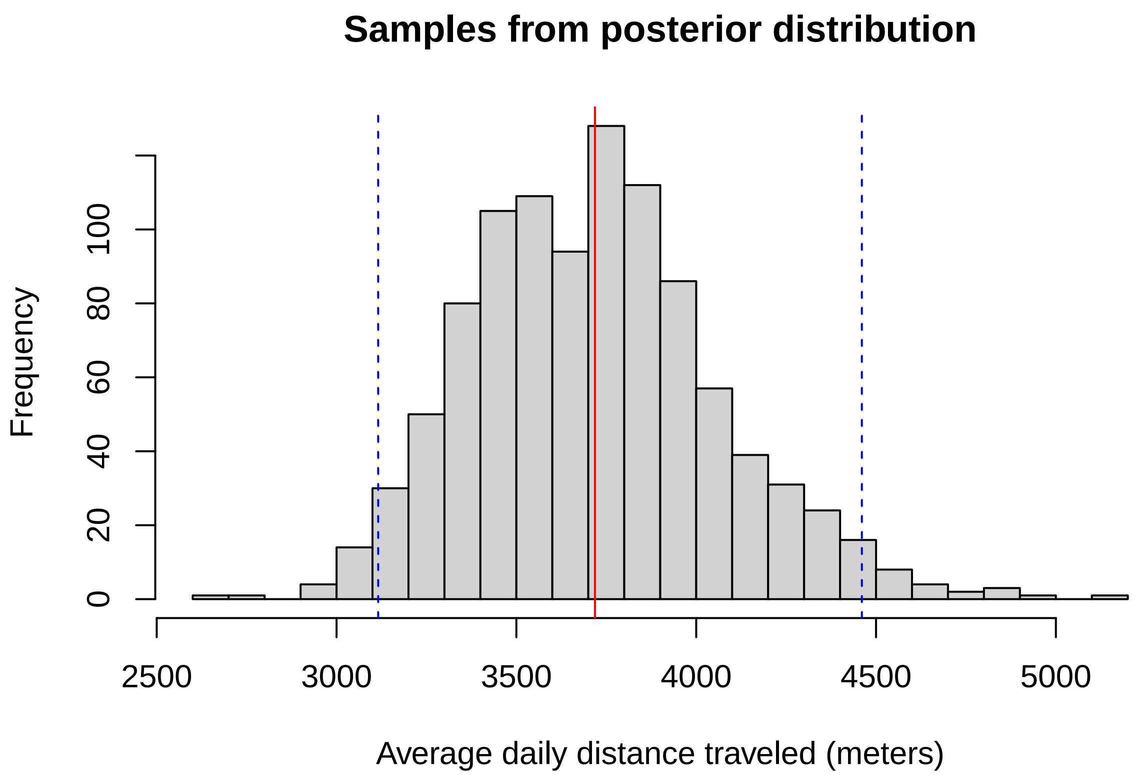

For inference at a different resolution, the above chunks must be edited.

Step 6) Repeat for individuals and study periods

We leave it to the practitioner to repeat this across individuals and chosen study periods, and to store the `all_paths` tables, and their desired derived quantity point estimates and variance estimates.

Speed up options

There are multiple options to increase run speeds.

Option A uses simple parallelization to fit both X and Y models simultaneously on your machine by using separate cores simultaneously. This can expect a modest ~50% speed up for only the model fitting step.

Option B offers the largest speed up (up to 10x), but requires slight reconfiguration of the computer and will depend slightly on the computer, making it more complicated.

Option C offers 2x or 3x speed up and is the most complicated to install, and requires reconfiguring the `tgp` package.

If speed is an important goal, combining option B and option C will have the best outcome.

Option A)

Simple parallelization of fitting the X and Y models. (This simple parallelization cannot be applied to prediction.)

```
library(parallel)
```

Replace fitModels chunk with:

```r
numCores <- detectCores() #the number of cores on your computer

#rewrite the tgp function as a function of either X or Y:
fit <- function(z) { btgpllm(X=individual$t.obs, Z= z,  bprior="b0", verb=0, pred.n = F) }
x_and_y <- list(individual$X, individual$Y)

## split into cores:
models <- mclapply(x_and_y, fit, mc.cores = numCores) #runs x and y fitting separately

names(models) <- c("X", "Y")
models$X #lets you grab the x_model
## In predict chunk, replace model_x with models$X and replace model_y with models$y
```

Option B)

Option B is to use a more efficient matrix algebra library within R. This will greatly speed up computations done within both stages of `tgp`. These changes must be made on the computer outside of R, but once this change is made then `tgp` fitting and prediction will automatically run faster in R every time, without any changes to code.

This can be done in the Accelerate framework on a mac, following installation instructions provided by Berkley statistics.

For windows, Intel's Math Kernel Library (MKL) can be used.

More details on Accelerate and MKL can be found in Robert Gramacy's Surrogates (2020).

Option C)

Option C requires uninstalling and reinstalling `tgp` with Pthreads. This will make `tgp` prediction automatically utilize parallelization. Details for this and reinstillation can be found in the `tgp` documentation appendix C.2.

References

Gramacy, R. B. (2007). tgp: an R package for Bayesian nonstationary, semiparametric nonlinear regression and design by treed Gaussian process models. Journal of Statistical Software, 19, 1-46. pdf

Gramacy, R. B., & Lee, H. K. H. (2008). Bayesian treed Gaussian process models with an application to computer modeling. Journal of the American Statistical Association, 103(483), 1119-1130. pdf

Gramacy, R. B. (2020). Surrogates: Gaussian process modeling, design, and optimization for the applied sciences. Chapman and Hall/CRC. book

Hooten, M. B., & Hefley, T. J. (2019). Bringing Bayesian models to life. CRC Press.

Hobbs, N. T., & Hooten, M. B. (2015). Bayesian models. Princeton University Press.
