# Supplementary material for: Treed Gaussian processes for animal movement modeling
Source: Ecol Evol. 2024 Jun 2;14(6):e11447. doi: 10.1002/ece3.11447 (PMC11144715; doi:10.1002/ece3.11447)
Supplement: Supplementary file 3 — Appendix S3 [file ECE3-14-e11447-s001.pdf]

Appendix S3: Additional information

1| Delta t choice

The Δt interval allows approximation of a continuous process by a finite number of discrete points. Including the uncertainty added by the Δt approximation in the total model uncertainty is an area for future research. Use of Δt prediction creates an approximately continuous trajectory, and thus any derived quantities obtained from the trajectory (e.g., distance traveled) will also be approximations. While Noonan et al., (2019) argues that Δt must be small enough to assume straight line travel between estimated points, we do not require this assumption and simply view the trajectory as approximate and resulting derived quantities as approximations. Because of this, comparing derived quantities computed using different Δt values is not recommended. When comparing results across studies or models, equal Δt values must be used.

A Δt approximation is used elsewhere in the continuous animal movement modeling literature and requires the user to select a Δt value (Hanks et al., 2011). We leave it up to the user to select a large enough Δt given the data available (for example, not expecting to produce inference at the 1-minute scale from daily GPS data). Smaller versus larger Δt also presents a tradeoff between accuracy and precision of the resulting estimates, and time of computation. Optimization of this tradeoff is also an area for future research.

For the purposes of our targeted practitioners, users can simply select a reasonable Δt (such as one hour to make inference on monthly derived quantities), fit the model and determine if computation times are reasonable for their needs, and adjust accordingly. The Δt parameter is simple to adjust within our framework. The value of precision versus computation time will depend on the research project and must be assessed by the user. The flexibility of our method allows users to set a very fine Δt at areas of interest (for example, an area of a potential road crossing) and a larger Δt at areas of less management interest.

2| Selection of MCMC sampling parameters

Within the `tgp` package, both fitting the model to the data and using the model to predict locations (the predict function) utilize Markov chain Monte Carlo (MCMC) sampling. MCMC methods are used to sample from the targeted posterior distribution, and there is a tradeoff between MCMC sample size (computation time and costs) and accuracy. For full understanding of MCMC sampling, we recommend knowledge of Bayesian statistics at the level of Hobbs and Hooten (2015). MCMC methods do require some evaluation by the practitioner and possible tuning of sampling parameters, however MCMC methods are now common in wildlife biology, and familiar to many practitioners.

In the `tgp` package, three MCMC sampling parameters are required to take a sample from the posterior, denoted by BTE and representing "burn in," "total," and "every." For example, BTE = (1000, 5000, 2) will remove the first 1000 samples as burn in, and thin by 2, resulting in an MCMC sample of size 2000. The `btgpllm` function for fitting the model (estimating model parameters) has default BTE settings of (2000, 7000, 2) and these do not need to be edited by the practitioner.

Sampling from the posterior predictive distribution of predicted locations, using the predict function, does require practitioner setting of the BTE sampling parameters. We recommend that

practitioners tune this based on their data and knowledge of MCMC methods. Code for examining trace plots and effective sample size is provided in Appendix S2. If tuning this value is infeasible, we recommend a MCMC sample size of 1000 using BTE = (2000, 12000, 10), however we have not tested this on multiple datasets. Practitioners may also avoid this tuning by choosing very large MCMC sample sizes at the cost of long run times.

3| Outliers and unusual movements

For telemetry data, we classify outliers (locations unexpectedly far from their temporal neighbors) into two types: location error outliers and process model outliers. Location error outliers arise from mechanical (or even data processing) errors and provide an unexpected location that is unrelated to where the animal actually was (beyond our expectations of telemetry device error). These outliers are common and familiar to practitioners working with telemetry data. They can be removed from datasets by setting a cut-off value for location or distance moved, or a cutoff for minimum number of recordings with a large movement, based on knowledge of study site and species behavior. These are removed before application of the TGP framework.

Process model outliers occur when the telemetry device is performing within its expected accuracy, but the animal has moved in some unusual way outside the pattern of the rest of the telemetry data. These outliers fall within the cutoffs that remove location error outliers, but still pose a challenge to modeling. They are considered outliers because they stand alone without a clear pattern. However, these points only appear as outliers because of the temporal fineness with which the data were collected. Remember that the model is seeking to express the true continuous-time path that underlies the discrete data points: there are no outliers in this true path. For example, a single data point that appears as a huge jump at a two-hour data scale could be easily explained and modeled if data were recorded at the minute scale.

It is important to recognize that the unusual movements present in process model outliers are likely very important ecologically, as is emphasized in the discussion. TGPs are able to detect these unusual movements when there are enough datapoints available (when the frequency of data collection is sufficiently smaller than the duration of the unusual movement). Figure 3 is an example of TGP successfully capturing an unusual movement, that was missed (treated as an outlier) by a non-treed model. However, all models, including TGP, are unable to model movements that occur at a finer temporal scale than the data is recorded at. This is why our model is not performing perfectly between the recorded datapoints at times 15:00 and 17:00 in Figure 3. In such a case there is not enough information present in the data, and the ideal would be to collect more data while this extreme movement is occurring.

While treed Gaussian processes perform very well at capturing extreme movement patterns that are present in the data, no modeling technique can compensate for a lack of data, and future work could investigate the scale at which data must be collected in order to detect a movement of interest. Knowledge of data requirements specific to the TGP model and the species and behavior of interest could be incorporated into adaptive sampling designs and this is an area for future research.

4| Telemetry device error

Incorporating and modeling telemetry device (e.g., GPS) error is common within animal movement models (e.g., Buderman et al., 2016). However, device error is not what wildlife practitioners are ultimately interested in. They instead wish to separate signal (actual movements of the animal) from noise (device error). As telemetry technology continues to improve, and errors become small enough to be relatively unimportant (e.g., ± 18 m accuracy), we believe too much focus on modeling device error detracts from more pressing scientific questions.

Treed Gaussian processes, like all models, do estimate noise in the data. In a Gaussian process, the nugget hyperparameter captures the discontinuity between the predicted and recorded location (Gramacy, 2020). While this may be interpreted quite literally as an estimate of device error, we would not use it as such. The model's estimate of the nugget incorporates *all* the "noisiness" in the data, most notably that created by process model outliers discussed in the previous section. This means we expect the nugget estimated from the data to be higher than the actual device error in most cases of animal movement data.

Again, this overestimation of noise is not a problem with the model, but one of not enough data. If the data is being interpreted by the model as noisy, it is because there are not enough datapoints to produce a clear signal. The model then interprets the process model outliers as noise, and inflates the nugget estimate and prediction variance. If there are enough data points, for example in Figure 3, the model can distinguish those unusual locations as signal instead of noise and model them as a separate node on the tree.

5| Treed partitioning for behavior classification

We recognize the clear connection between treed partitioning of movement and behavior classification, as well as the ample work already done on modeling behavior states, and believe there could be promising future work on integrating the two. Identifying likely behaviour states from telemetry data is valuable to practitioners, and movement models focused on classifying behaviors are common in the literature (Edelhoff et al., 2016; Wang, 2019). While our work focused on the ability of TGPs to predict the underlying movement trajectory, the partitioning capabilities of TGPs hold promise for data-driven classification of movement (Broderick & Gramacy, 2011). Harnessing the classification power of TGPs to study behaviour states is a natural direction for future research.

However, it is worth emphasizing that our model and inferential framework in its current form is not designed for explicit behavior inference. The current avenue for behavior classification using the TGP modeling framework would be to either define movement characteristics of behavior states using specific derived quantities (e.g., speed, turn angles) or use estimates of derived quantities to inform practitioner decisions on behavior state classification. The current avenue for classification of behavior states within the derived quantity portion of the inferential framework emphasizes that assumptions are not being made by the TGP model regarding behavior, and that these assumptions and definitions must be made by a user wishing to classify behavior states.

Broderick, T., & Gramacy, R. B. (2011). Classification and categorical inputs with treed Gaussian process models. *Journal of Classification*, 28(2), 244-270.

Edelhoff, H., Signer, J., & Balkenhol, N. (2016). Path segmentation for beginners: an overview of current methods for detecting changes in animal movement patterns. *Movement Ecology*, 4(1), 1-21.

Wang, G. (2019). Machine learning for inferring animal behavior from location and movement data. *EcologicalInformatics*, *49*, 69-76.

6| Population-level modeling and model splits

The treed aspect of the TGP modeling framework is conducive to the concept of a complete or full model that models and includes all information in the dataset. This full model would simultaneously model information from both X and Y directions, and information from all individuals in the dataset. Splits in the treed partitioning would then separate sub-models pertaining to individuals, or groups of individuals, that travel together. This is a possible basis for population level modeling using the TGP framework. If we instead model individual animals separately, we are assuming they move independently. This may be functionally true for some species and individuals, but not others.

With current computation capacities, fitting the full model to all aspects of the dataset would be computationally preventative for many datasets and practitioners. In our framework, we chose to separate individuals and lose the possible information gained from modeling multiple individuals' movements simultaneously. This allowed our first TGP applications to be computationally tractable and accessible.

When fitting separate models to different data, we assume independence of those processes and accept any loss of information from possible dependence of those processes. Deciding where to split data and models is therefore a balance between computational simplicity and possible loss of information. In addition to modeling individuals separately, we chose to model X and Y directions separately, making the simplifying assumption that movement in one direction is independent of movement in the other. This assumption is common in the continuous-time animal movement modeling literature; see Hooten and Johnson (2017) equations 1-2, see also Ch. 6 (e.g., Eq 6.65) of Hooten et al. (2017). Practically, by assuming X-directional and Y-directional movements are independent, we can cut our model fitting time in half by fitting separate `btgpllm` models to X data and Y data, allowing for parallelization.

Modeling can be further split based on research questions and ecological knowledge of the system and animals. If an animal has data that spans a long time period, for example years, the researcher may wish to divide data into known life history seasons for the species under the assumption that the animal behaves and moves differently in these seasons. This will greatly speed up computation. However, like with individuals, the full model would model all the seasons together and allow the treed partitioning to make seasonal divisions. In this latter case, the seasonal divisions are made based on the data, as opposed to life history cutoff dates. For example, a researcher may use a nesting season start date from the literature of June 15th to divide the data and fit a model for the nesting season, but in a full model the TGP may find that the bird doesn't begin nesting characteristic movements until June 19th, and thus the full model makes a treed split there.

Splitting at time periods also assumes independence of an individual animal's movements at different periods. Though an animal's movements are not truly independent at different times of its life as they still come from the same individual, this may be a valuable simplifying assumption for practical modeling purposes and reduce computation costs. Splitting within time periods will also increase ease if research questions are already framed within the scale of these time periods. Generally, model splitting decisions will be up to the practitioner's discretion and depend on the amount of data, computational constraints, research questions, and the known life history and patterns of the species.

7| Tree breaks and discontinuities

Treed Gaussian processes have been recognized for their ability to model discontinuities in data, and this strength applies directly to the abrupt changes present in animal movement data (Gramacy & Lee, 2008). We recognize that physical movement is not in reality discontinuous. In using TGPs we break from the mechanistic models most frequently used to model telemetry data based on the physical laws of movement and instead present a phenomenological model focused on predictive accuracy and increased model fitting abilities.

Figure 3 demonstrates the ability of TGPs to model abrupt transitions in patterns of movement and location. Our recorded GPS data show that the lesser prairie-chicken in Figure 3 traveled over 14 km in the two-hour interval between recordings at hours 15:00 and 17:00 on 2014-06-06, a movement that the Gaussian process model fails to capture. In the TGP model, this extreme change is treated as a "break" in the model: a shift to a different node of the treed partitioning. Having such a break implies discontinuous movement, going against natural instincts of continuous-time movement: as Δt approaches zero, a bird cannot actually travel 14 km between consecutive time points (e.g., infinite velocity of a bird).

However, we continue with our phenomenological (TGP) approach to modeling and argue that the model is performing as we would hope to meet our predictive and inferential goals. Though this discontinuous movement may be viewed as a mechanistic failing, mechanistic accuracy is not our goal. Our phenomenological model is performing prediction as desired for that individual's extreme movement. If we step back from modeling, it is clear that the GPS data we have are incapable of accurately telling us where the bird was at time 16:00 during such an extreme and unprecedented movement behavior. Thus, the phenomenological model makes predictions in either of the two data clumps at time 16:00. Our model does not provide information that was not present in the data, but it does estimate what we need to obtain our derived quantities and appropriate uncertainty.

It is worth emphasizing that this discontinuity of the predicted movement trajectory does not mean that this is no longer a continuous-time animal movement model. The model is assuming a continuous-time function (the Gaussian process) as the underlying function describing the animal movement within the partitions, thus retaining the properties of a continuous-time model. Additionally, the treed Gaussian process model that creates disjunct transitions between partitions is defined across continuous time. This means it can make predictions of location at any time, making it a continuous-time animal movement model.

8| The future of animal movement analysis

As telemetry data continues to improve and device error continues to decrease, our data itself will approach the true trajectory. This will decrease the importance of the modeling steps in Figure 1, while increasing the importance of the inference steps. Our proposed TGP modeling framework is a statistical stand-in until the future time when we have data on the complete animal trajectory. However, even when TGPs are replaced by improved machine learning models, and eventually replaced by near-continuous animal telemetry data, the inferential framework outlined in Figure 1 will still hold. Utilizing machine learning to nearly automate the process of estimating continuous trajectories from discrete telemetry data allows animal movement modeling to follow technology's progression towards continuous telemetry data.
