# Supplementary material for: Treed Gaussian processes for animal movement modeling
Source: Ecol Evol. 2024 Jun 2;14(6):e11447. doi: 10.1002/ece3.11447 (PMC11144715; doi:10.1002/ece3.11447)
Supplement: Supplementary file 4 — Appendix S4 [file ECE3-14-e11447-s004.pdf]

Appendix S4: Example applied trajectory transformations

This appendix provides a detailed example for estimating average daily total distance traveled across one month for one individual, starting from a sample of outputted trajectories. It continues with a demonstration of population level inference. Tables of example values are provided, similar to how they would appear in R (see Appendix S2 for full R code, see Appendix S1 for full definitions of notation).

Period = 1 month
Δt = 1 hour
MCMC sample size = 1000

<u>MCMC sample of locations **S**:</u>
$S_{1,t}^{(k)}$ = the kth MCMC sample at time *t* in the easting direction (units = UTM)
$S_{2,t}^{(k)}$ = the kth MCMC sample at time *t* in the northing direction (units = UTM)

t = hour	$S_{1,t}^{(1)}$	$S_{2,t}^{(1)}$	$S_{1,t}^{(2)}$	$S_{2,t}^{(2)}$	…	$S_{1,t}^{(1000)}$	$S_{2,t}^{(1000)}$
0	483382.5	4144834	483213.4	4145334		483420.9	4144674
1	482812.7	4145860	483149.9	4145201		483121.6	4145108
…							
744	500295.2	4128498	500221.0	4128543		500278.8	4128570

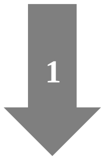

Use distance formula to compute hourly displacement between each Δt set of points

<u>MCMC sample of displacements:</u>
$D_t^{(1)}$ = the kth MCMC sample of hourly displacement at time *t* (units = meters/Δt = meters/hour)

t = hour	$D_t^{(1)}$	$D_t^{(2)}$	…	$D_t^{(1000)}$
1	1173.41451	148.16156		527.19819
2	791.75742	168.24507		52.77138
…				
744	63.811221	61.99200		180.248800

2 *Sum within each column's day*

<u>MCMC sample of daily total distance traveled:</u>

$D_{day}^{(k)}$ = the kth MCMC sample of total daily distance traveled at day *day (*units = meters/day)

t = day	$D_{day}^{(1)}$	$D_{day}^{(2)}$	…	$D_{day}^{(1000)}$
24 – day 1	9884.867	7074.448		6867.132
48 – day 2	9365.736	9002.061		7493.807
…				
744 – day 31	1969.755	2986.166		2441.949

3 *Take column averages*

<u>MCMC sample of average daily distance traveled in that month:</u>

$\overline{D}^{(k)}$ = the kth MCMC sample of average daily distance traveled (units = meters/day)

	$\overline{D}^{(1)}$	$\overline{D}^{(2)}$	…	$\overline{D}^{(1000)}$
Whole season	10143.601	9038.150		9691.998

Extension to Population:

<u>Population level inference on average daily distance traveled across a *population* of n animals:</u>

Compute the above vector (dimension = 1 x 1000) for each of the n individual animals.

Row bind these vectors into a table of dimension = n x 1000

Take column averages – this ensures MCMC samples are aligned. See Hobbs and Hooten (2015) chapter 8.3 for more details on aligning derived quantities.

This results in a 1x1000 vector of samples from the posterior distribution of population average daily distance traveled.
